# Natural remedies proposed for the management of diabetic retinopathy (DR): diabetic complications

**DOI:** 10.1007/s00210-025-03866-w

**Published:** 2025-02-15

**Authors:** Emad Gamil Khidr, Nourhan Ibrahim Morad, Shymaa Hatem, Ahmed M. El-Dessouki, Ahmed F. Mohamed, Riham A. El-Shiekh, Mohamed S. Abd El Hafeez, Heba R. Ghaiad

**Affiliations:** 1 Biochemistry and Molecular Biology Department, Faculty of Pharmacy (Boys), Al-Azhar University, Cairo, Egypt; 2 Department of Pharmacognosy and Natural Products, Faculty of Pharmacy, Menofia University, Menofia, Egypt; 3 Department of Pharmaceutics and Pharmaceutical Technology, Faculty of Pharmacy, Future University in Egypt, Cairo, Egypt; 4 Pharmacology and Toxicology Department, Faculty of Pharmacy, Ahram Canadian University, 6Th of October City, Giza, 12566 Egypt; 5 Department of Pharmacology and Toxicology, Faculty of Pharmacy, Cairo University, Cairo, 11562 Egypt; 6 Faculty of Pharmacy, King Salman International University (KSIU), Ras Sedr, South Sinai, 46612 Egypt; 7 Department of Pharmacognosy, Faculty of Pharmacy, Cairo University, Kasr El-Aini Street, Cairo, 11562 Egypt; 8 Department of Pharmacy, Kut University College, Al Kut, Wasit, 52001 Iraq; 9 Department of Pharmacognosy, Faculty of Pharmacy, Egyptian Russian University, Cairo-Suez Road, Badr, 11829 Egypt; 10 Biochemistry Department, Faculty of Pharmacy, Cairo University, Kasr El Ainy St., Cairo, 11562 Egypt

**Keywords:** Diabetic retinopathy (DR), Diabetic outcomes, Natural products, Diabetes mellitus (DM), Diabetic complications

## Abstract

Diabetic retinopathy (DR) represents a significant and serious complication associated with diabetes mellitus (DM), often resulting in considerable visual impairment or even blindness. The intricate pathological processes underlying DR complicate the effectiveness of current treatment modalities. Studies have highlighted the potential of natural products in the treatment of DR via several beneficial effects including anti-inflammatory, antioxidant, anti-neovascular, and anti-apoptotic properties. Flavonoids, saponins, saccharides, and alkaloids exhibited various beneficial effects in DR in in vivo and in vitro studies. However, the clinical utilization of these natural compounds is hindered by issues such as inadequate specificity, low bioavailability, and potential toxicity. Therefore, there is a pressing need for rigorous clinical studies to confirm the efficacy of natural products in preventing or mitigating the progression of DR.

## Introduction

Diabetic retinopathy (DR) is a significant complication of diabetes mellitus (DM), currently impacting approximately 103.12 million individuals globally. By the year 2045, it is projected that DR will impact approximately 1.6 million individuals globally (Teo et al. 2021). DR remains a major contributor to vision loss in both developed and developing nations, imposing considerable economic burdens on healthcare systems, especially as the disease progresses from early to advanced stages (Orji et al. 2021). DR is categorized into two stages: non-proliferative diabetic retinopathy (NPDR) and proliferative diabetic retinopathy (PDR) (Mrowicka et al. 2024). NPDR, the initial stage of DR, may not show any noticeable symptoms, but the funds display pathological changes, including microaneurysms, hemorrhages, cotton-wool spots, and hard exudates. PDR represents a more advanced stage of NPDR and is primarily linked to ongoing hyperglycemia and poor glycemic management (Gangwani et al. 2016). The specific mechanisms and pathophysiology of DR are not fully understood. The standard treatment for preventing vision loss in DR patients is panretinal photocoagulation; however, this laser procedure can lead to various ocular complications, including damage to visual acuity and visual field. Other treatment options present their own challenges and side effects; for instance, intravitreal injections of anti-vascular endothelial growth factor (VEGF) do not completely halt DR progression, and repeated treatments increase the risk of endophthalmitis (Wykoff 2017). Advancements in herbal medicines are offering new strategies for the management of DR. In vitro and in vivo studies have supported evidence for the value of polyphenols as potential nontoxic agents to avoid pathology from advancing. Phytocompounds, i.e., flavonoids, have a potential to prevent the disruption of the blood retinal barrier, improve the oxidative state, decrease the release of proinflammatory mediators, and prevent the reduction in retina thickness by reducing apoptosis and neurodegeneration (Matos et al. 2020). Given their limited bioavailability and concentration in meals, developing local delivery systems would be a viable option for using them as an effective therapeutic (Rodríguez et al. 2019). This review aims to provide an overview of the current understanding of DR’s pathophysiology, available therapeutic strategies, and the potential of natural products in the management of DR.

## Epidemiology

DR is a significant cause of visual impairment worldwide, particularly affecting individuals aged 25 to 74 years. Visual loss from DR may result from complications such as macular edema (ME), retinal detachment, hemorrhage, or neovascular glaucoma. As DR often remains asymptomatic until advanced stages, regular screening is crucial to detect disease early and prevent progression (Flaxel et al. 2020). Diabetic macular edema (DME) may develop at any stage of DR, from mild non-proliferative to proliferative disease. These classifications are essential for guiding treatment strategies, though each patient’s progression and symptoms are unique, requiring individualized care (Wong et al. 2016).

Globally, the prevalence of DR is estimated at 34.6% (Yau et al. 2012). The incidence is comparable between type 1 and type 2 diabetes, with higher rates observed in some ethnic populations, though these trends may vary regionally (Sivaprasad et al. 2012).

Key risk factors for DR include hyperglycemia, where a 1% reduction in HbA1C levels is associated with a 35% decreased risk of DR and significant reductions in progression and vision-threatening complications (Schreur et al. 2018; Ng et al. 2019). The duration of diabetes is also critical, with 50–90% of patients developing DR after 20 or more years (Song et al. 2018). Hypertension significantly impacts progression risk, with a 10 mm Hg reduction in systolic blood pressure associated with a 40–50% reduced risk (Gallego et al. 2008). Additional factors include nephropathy, dyslipidemia, obesity, and certain genetic predispositions. These risk factors highlight the importance of comprehensive management in preventing DR progression (Wong et al. 2016).

## Diagnosis

DR is a potential complication in any diabetic patient and regular screening is needed to ensure early treatment and prevent progression as well as vision loss. DR should be suspected in any diabetic patient with visual impairment especially when risk factors are present (Flaxel et al. 2020; 2025).

Retinal exams are necessary for diagnosis. Mild NPDR presents microaneurisms without other retinal findings. Moderate NPDR presents with microaneurisms plus additional signs of vascular abnormalities that do not meet criteria for severe NPDR. Severe NPDR presents with microaneurisms plus any of severe intraretinal hemorrhages with microaneurisms in each quadrant, definite venous beading in ≥ 2 quadrants or moderate intraretinal microvascular abnormalities in ≥ 1 quadrant (Flaxel et al. 2020).

PDR presents with characteristics of severe NPDR plus neovascularization and/or vitreous/preretinal hemorrhage. High-risk PDR has any 3 of neovascularization at any location, neovascularization at or near optic disc, moderate or worse neovascularization, and vitreous/preretinal hemorrhage (Flaxel et al. 2020).

DME presents with any of the following: thickening of retina within 500 µm of center of the macula, hard exudates (lipid deposits) within 500 µm of center of the macula when associated with adjacent retinal thickening and ≥ 1 zones of retinal thickening of 1 disc area or larger in which any portion of thickening is within 1 disc diameter of the center of the macula (Flaxel et al. 2020).

Retinal examination to make the diagnosis may include direct or indirect fundoscopy, fundus photography, slit lamp biomicroscopy, and undilated gonioscopy (Fenner et al. 2018).

## Pathogenesis

DR is a severe complication of diabetes, primarily resulting from prolonged hyperglycemia, and is the leading cause of vision impairment among working-age adults globally (Al Zabadi et al. 2022). The condition is marked by microvascular damage, retinal ischemia, and hypoxia, contributing to the progression of retinal disease (Lange et al. 2021). The pathogenesis of DR is driven by several mechanisms, including oxidative stress, inflammation, advanced glycation end products, and angiogenesis. Disruptions in metabolic pathways, such as the polyol pathway and insulin resistance, further exacerbate retinal damage (Tang et al. 2023).

Oxidative stress plays a critical role in the early stages of DR by promoting leukocyte adhesion to retinal capillaries, while inflammation, advanced glycation end products, and polyol accumulation also contribute to vascular dysfunction (Mrugacz et al. 2021). These processes are interconnected, with circulating proinflammatory and proangiogenic factors further exacerbating retinal damage. Hormonal imbalances, including those related to insulin and gut hormones, also influence DR progression, and recent studies have shown that gut microbiota may play a significant role in regulating inflammation and oxidative stress in diabetic patients (Kang and Yang 2020; ValdezGuerrero et al. 2021; Bany Bakar et al. 2023). Additionally, oxidative stress-induced cholesterol accumulation in the retina leads to retinal hypoxia, inflammation, and blood–barrier disruption (Varghese and Ali 2021; Aldosari et al. 2022; Xu et al. 2024).

### Role of nutritional factors

Dietary factors significantly impact the development and progression of DR by influencing oxidative stress, inflammation, and endothelial function (Pellegrini et al. 2020). High-glycemic diets are linked to an increased risk of microvascular disease, while antioxidants and specific nutrients, such as vitamin C, omega-3 fatty acids, and monounsaturated fatty acids, have shown potential in reducing the risk of DR (Shah et al. 2022; Sasaki et al. 2023). Studies suggest that obesity and insulin resistance contribute to inflammation, further promoting DR progression (Sachan et al. 2020). Targeted dietary interventions, alongside lifestyle changes, may help reduce the risk of DR in individuals with diabetes (Bryl et al. 2022).

### Impact of gut microbiota

Emerging evidence highlights the role of gut microbiota in the pathogenesis of DR. Gut bacteria influence metabolic and inflammatory processes, which can impact retinal health. These microorganisms produce beneficial metabolites like butyrate, which play a role in immune regulation and may protect against retinal damage (Huang et al. 2021b).

The gut-eye axis, mediated by the vagus nerve, suggests that gut health may influence ocular inflammation and DR progression. Research on gut microbiota interventions, such as fecal microbiota transplants, is showing promise in modulating retinal inflammation and improving outcomes in DR patients (Abdelrahman et al. 2022; Su et al. 2022).

### Contribution of genetic factors

Genetic predispositions are a key factor in the development and progression of DR. Studies have shown that individuals with a family history of diabetes are at a higher risk of developing DR, and the heritability of DR is estimated to be 23% (Xiong et al. 2020). Genetic variations in inflammation pathways, glucose metabolism, and vascular health are linked to increased DR risk (Yang and Liu 2022). Understanding these genetic factors is crucial for identifying personalized treatment strategies, as individuals with specific genetic profiles may respond differently to therapies (Chen et al. 2022).

Advances in genetic research, including genome-wide association studies, are helping to uncover new therapeutic targets for DR, offering hope for more effective precision medicine approaches (Antonetti et al. 2021; Georgiou et al. 2024).

## Complications

DR is also associated with several complications that worsen the prognosis, and once it is affected, it would be very difficult to reverse it. Early detection and management are very important to prevent these complications, including diabetic macular edema, which is the leading cause of visual loss in DR. Proliferative DR morbidity increases with the presence of vitreous hemorrhage, neovascular glaucoma, tractional retinal detachment, and blindness (Zhang et al. 2022a, b, c). It is important to separate these two entities to form different surgical plans to reduce the risk of retinal detachments and PDR-associated blindness. Blindness occurs in severe cases due to vitreous hemorrhage, tractional retinal detachment, or neovascular glaucoma. Vitreous hemorrhage and cataract are the leading causes of visual impairment. Intraocular pressure elevation, corneal pathologies, and changes in the angle of the anterior chamber are complications that can be expected because of the effect of PDR (Shaikh et al. 2023).

Diabetic patients have a 25-fold increased risk of blindness from vitreous hemorrhage, as well as tractional retinal detachment, pre-retinal, and pre-macular hemorrhages associated with the proliferation of new abnormal retinal vessels (Gáll et al. 2024). Visual conditions affecting diabetic patients should not be underestimated, and these sight-threatening conditions can have a profound qualitative or even suicidal impact. Vision loss due to the retraction stages of DR may cause adjustments in psychosocial and lifestyle factors because of their increased reliance on others (Oboh and Precious 2024). Vitrectomy is a treatment used as a salvage with the aim of visual recovery over a short or medium-term period. Overall, visual outcomes are associated with increased duration of diabetes. Therefore, improvement in education and early screening for DR for diabetic patients and healthcare providers is important. In developing the concept to prevent and treat diabetes, points of multifactorial attitudes, including gene polymorphism, consist of the factors that are currently under consideration in the era of a new paradigm of maintenance healthcare (Wykoff et al. 2021).

The complications of the aforementioned disease are varied and complex. Most morbidity arises from elevated glucose levels. The initial non-proliferative form exhibits microaneurysms known as dot and blot hemorrhages, hard exudates, and retinal thickening. In a proliferative form, vascular deregulations lead to retinal ischemia and angiogenesis, which can appear as cotton wool spots, venous bleeding, or neovascularization (Rajpal et al. 2020). Angiogenesis will not only occur in the retina but can also spread to the surface of the posterior case. Renin-angiotensin system activation can be represented by polymorphism, which will induce aggregation of fibroblast tissue and then trigger endothelial cellular apoptosis. Neovascularization may induce vitreous hemorrhage and tractional retinal detachments, leading to visual impairment, including blindness. Psychological suffering from the inability to perform normal activities caused by dark vision weight can lead to efforts to increase their vision magnitude (Chalise and Hale 2024). Treatment for DR, which currently focuses on intravitreal anti-VEGF treatment and surgical intervention, reduces DR complications. Networking responses in systemic pathology may cause edema in the retina, leading to hypoxia from high activity of COX-2. Monocyte recruitment and platelet adhesion also increase (Garvin et al. 2021). For a surgeon, understanding the complications involved in DR, a common primary retinal pathology, is essential so that management can be carried out at the right time. The increase in the number of patients with diabetes worldwide is also increasing the demand for assistance, particularly for children with diabetes. In addition, a team comprising sub-specialists, endocrinologists, and dietitians can not only manage but also reverse the condition, offering better management.

### Macular edema

Macular edema (ME), characterized by fluid leakage from perifoveal capillaries, is the most prevalent complication of DR. It results in vision impairment by involving the central retina in the macula, which has evolved from the primary site of visual centers (Lindner et al. 2022). Perifoveal capillary blood flow plays a key role in photoreceptor metabolism, and their leakage leads to photoreceptor destruction due to factors such as lack of oxygen, disrupted blood flow, and impaired vision. The resultant increased vascular permeability from chronic inflammation induces a sustained release of VEGF, which leads to prolonged transcription changes in the expression of multiple endothelial cell genes, resulting in fluid accumulation near and in the fovea (Matsuno et al. 2022). ME is a characteristic feature in diabetic patients with a reduction of central visual function, and its progression is prevalent in all forms of diabetes. In clinical practice, approximately 30% of individuals in their medical records, usually those with long-standing diabetes and often with nephropathy or hypertension, have ME. The major risk factors for the development of ME during the progression of DR include uncontrolled chronic hyperglycemia, prolonged duration of diabetes, and in men, lower effective insulin use, body mass index, LDL levels, and triglycerides, as well as dyslipidemia in some reports. It is directly linked to and also triggered by long-lasting hypertension, which not only obstructs major blood flow in foveal capillaries but also induces the complement system and protein kinase pathways to block the activation of transporter KCC, which is essential for liquid absorption by perifoveal Müller glia (Han et al. 2021). Measuring the transmembrane proteinases in blood and vitreous humor of the patients, such as the junction-adhesion molecule-like might reflect the destruction of endothelial tight junction–adhesion proteins. Reactive microvascular disease caused by inhibition of nitric oxide increases retinal vascular leakage by disrupting the barrier between the extracellular matrix and retina. The risk factors for DR in the early stage of type 1 diabetes include younger age, poor healthcare access, increasing puberty, length of diabetes, and the potential possibility of pregnancy (Ferrat et al. 2020).

### Neovascular glaucoma

Neovascular glaucoma (NVG) is a dangerous complication that can develop in the severe forms of DR. At the basis of its pathogenesis, there is the process of retinal ischemia with the subsequent neovascularization of the anterior segment of the eye. Neovascularization is a compensatory mechanism resulting from the increasing level of VEGF. The formation of abnormal blood vessels on the surface of the iris leads to their closure, the obstruction of the posterior chamber angle, and eventually fibrous tissue and closure of the angle. This, combined with extensive neovascularization, results in elevated intraocular pressure with irreversible damage to the optic nerve. The clinical presentation of NVG is extremely aggressive and difficult to pursue. Pain is referred to as orbital pain or pain in the supraorbital region. Subjects affected claim to have experienced an acute loss in visual acuity, associated with a gradual loss of vision, frequent redness, and tearing in the eye (Shazly and Latina 2009; Takayama et al. 2019).

Neovascular glaucoma is difficult to manage and poses multiple therapeutic problems. The goal of treatment is to reduce the level of intraocular pressure and to reduce neovascularization. Various types of pharmacological treatments have been discussed, but surgical treatment is the only ultimate option in progressive cases. Bearing in mind the complexity of the onset mechanism and the seriousness of the clinical signs, NVG must be, if possible, subjected to a timely form of prevention and a multidisciplinary approach, necessarily designed following a careful assessment of the risks and benefits. The prevention and prompt and correct medical–pharmacological treatment are aimed at preserving visual function, sometimes in a very threatening situation (Senthil et al. 2021).

## Therapeutic targets in the management of DR

The blood-retinal barrier (BRB) held a fundamental function in preserving retinal homeostasis via controlling the exchange of molecules, ions, and cells between the blood and the retina and constitutes the first line shield and the initial barrier for the retinal cells against the external detrimental agents (Yang et al. 2020; Zhang et al. 2023). BRB consists of a complex multicellular structure of two components. First, the inner BRB is composed by barrier-forming tight intracellular junctions of capillary endothelium, supported by pericytes and glial cells like Müller cells and astrocytes (Rudraraju et al. 2020). Then, there is the outer BRB which is essentially maintained by retinal pigment epithelial (RPE) cells that regulate solute transport across the sub-retinal space (O’Leary and Campbell 2023).

### Blood retinal barrier breakdown

In DR, chronic hyperglycemia generates reactive oxygen species (ROS) which directly damages endothelial tight junction proteins, resulting in increased permeability of inner BRB, vascular leakage, and subsequent retinal pathology (Eshaq et al. 2017). Under diabetic conditions, ischemia and hypoxia upregulate VEGF, particularly via stabilization of hypoxia-inducible factor-1α (HIF-1α) (Thangarajah et al. 2010). VEGF has been reported to increase the proliferation and migration of endothelial cells which will subsequently leads to increased vascular permeability and DME (Ahuja et al. 2019). In addition, overexpression of VEGF activate protein kinase C (PKC) that subsequently induce occludin phosphorylation, leading to its internalization which will subsequently lead to tight junction disassembly and breakdown of BRB (Reddy et al. 2024). Consequently, retinal endothelium secretes cell adhesion molecules (CAMS) including intercellular adhesion molecule 1 (ICAM-1) and vascular cell adhesion molecule 1 (VCAM-1). On the luminal side, CAMS act as anchors so the circulating leukocytes can attach to the endothelium and secrete proinflammatory cytokines (Yang et al. 2020; O’Leary and Campbell 2023). Eventually, the accumulation of cytotoxic mediators will create a local pro-inflammatory milieu that can further weaken the BRB integrity (Yang et al. 2020; Zhang et al. 2023). Anti-VEGF agents are effective in blocking the cycle of BRB breakdown VEGF inhibitors, as well as anti-inflammatory agents that reduce cytokine and leukocyte-mediated damage, preserving BRB (Patel et al. 2024).

### Neovascularization

Neovascularization in DR is a maladaptive response to retinal hypoxia, primarily driven by the overexpression of VEGF and inflammatory mediators (Pei et al. 2024). Neovascularization is a prevalent pathological hallmark of DR that indicates the growth of dysfunctional and newly formed vasculature. From a clinical perspective, neovascularization is depicted on the retinal surface by tiny vasculature networks that extends down into the vitreous cavity (Nian et al. 2021). In DR, retinal ischemia induces the release of several growth factors, including VEGF and platelet-derived growth factor (PDGF), to promote blood vessel formation. PDGF specifically stabilizes newly formed blood vessels by attracting pericytes which are critical for vascular maturation and stability. VEGF interactions with receptors VEGFR1/VEGFR2 and co-receptors like neuropilin-1 drive vascular leakage and angiogenesis (Wiszniak et al. 2021). Anti-VEGF therapies, such as bevacizumab, reduce neovascularization but require long-term treatments (Spooner et al. 2021; Boscia et al. 2024). Additionally, PDGF inhibitors prevent neovascular proliferation. Furthermore, profilin1 (Pfn1)-actin interaction inhibitors (e.g., C74) play a significant role in regulating neovascularization by targeting the actin cytoskeleton dynamics that are crucial for endothelial cell migration and angiogenesis (Reddy et al. 2024).

### Leukostasis

Leukostasis refers to the obstruction of retinal microvasculature by leukocytes followed by retinal endothelial necrosis in DR. During the early stages of DR, leukocyte-endothelial interactions trigger an escalating inflammatory response, leading to the further accumulation and entrapment of leukocytes within the retinal microvasculature followed by the blockage of blood flow in the narrowed retinal vessels (Wal et al. 2024). In advanced stages of DR, the systemic neutrophil count increases causing overexpression of myeloperoxidase and overproduction of hydrogen peroxide (Reddy et al. 2024). Endothelial ICAM-1 and VCAM-1 upregulation fosters leukocyte adhesion, leading to capillary occlusion and cytokine release. Moreover, VEGF exacerbates leukocyte-endothelial interactions and amplifies oxidative stress. Increased adhesion of leukocytes to retinal vessels exacerbates inflammation and vascular occlusion (Wiszniak et al. 2021). Blocking leukocyte-endothelial interactions with VEGF inhibitors and anti-inflammatory agents reduces leukostasis and preserves vascular integrity. VEGF blockers and ICAM-1/CD18 inhibitors reduce leukocyte adhesion and endothelial dysfunction, alleviating microvascular occlusion (Li, Zhao and Sun 2024a, b, c, d; Reddy et al. 2024).

### Retinal inflammation

Retinal inflammation is essentially correlated with DR severity. Nonetheless, up till now, no single proinflammatory mediator has been fully linked with DR progression. Subclinical inflammation has been associated with pericyte and endothelial cell loss, and thickening of the retinal basement membrane in DR (Wang et al. 2024b). Inflammation in DR is known to persist throughout the disease progression, from the early stages to the advanced, vision-threatening phases. Chronic inflammation, driven by cytokines including TNF-α, IL-6 disrupt vascular homeostasis (Rojas et al. 2024). Moreover, ROS generation and endoplasmic reticulum (ER) stress amplify inflammatory responses, leading to tissue damage. Anti-inflammatory agents (e.g., corticosteroids, cannabinoids) have been proven to be beneficial in DR via suppressing cytokine signaling.

### Oxidative and nitrosative stress

Oxidative stress underpins DR pathology. In DR, chronic hyperglycemia generates ROS through glucose autooxidation, polyol pathway, the formation of advanced glycation end products (AGEs), the activation of PKC, and nicotinamide adenine dinucleotide phosphate oxidase. Moreover, VEGF signaling has been reported to exacerbate oxidative damage. ROS directly damages endothelial tight junction proteins, leading to increased permeability of inner BRB, vascular leakage, and subsequent retinal pathology (Reddy et al. 2024). In fact, excessive ROS and peroxynitrite impair mitochondrial function, oxidize proteins, and activate apoptosis pathways (Ristori 2021; Reddy et al. 2024; Wal et al. 2024). Furthermore, peroxynitrite disrupts VEGF and NGF signaling, leading to vascular and neuronal damage. Peroxynitrite also nitrates tyrosine residues, impairing VEGF and NGF signaling, leading to endothelial apoptosis and neurodegeneration (Flores et al. 2023; González et al. 2023).

Therefore, strategies to prevent the destructive effects of oxidative and nitrosative stress have been postulated as possible treatment options for DR. Several studies showed that reduction of oxidative stress with antioxidant treatment could prevent diabetes-induced retinal vascular dysfunction and increases in VEGF expression (Andrés-Blasco et al. 2023; Gao et al. 2023). Novel approaches target peroxynitrite as a critical mediator of VEGF-driven angiogenesis. FeTPPs, which is a ferric porphyrin complex capable of decomposing peroxynitrite to produce nitrate, has been reported to restore pro-survival signaling and prevent neuronal and vascular damage caused by nitrative stress. Neutralizing peroxynitrite with FeTPPs or selective blockade of tyrosine nitration with epicatechin prevented endothelial cell death (Liu et al. 2024).

### Polyol pathway

The polyol pathway, driven by aldose reductase, exacerbates oxidative and osmotic stress in DR. The polyol pathway comprises the aldose reductase (AR) enzyme which reduces glucose to sorbitol using NADPH as a cofactor, and sorbitol dehydrogenase (SDH) enzyme, which converts sorbitol to fructose using NAD + as a cofactor. In retina, sorbitol is known to accumulate osmotic stress that damages retinal endothelial cells and pericytes. Moreover, sorbitol production depletes NADPH and increases oxidative stress (Dănilă et al. 2024). Blocking AR, which is the rate-limiting enzyme of polyol pathway, by inhibitors like Fidarestat and Sorbinil has been shown to reduce sorbitol-induced vascular damage (Dănilă et al. 2024; Reddy et al. 2024).

## Current treatment options for DR

DR remains the leading cause of vision loss among working-age adults, emphasizing the need for effective and comprehensive therapeutic strategies (Kropp et al. 2023). Current treatment modalities aim to address both the pathological mechanisms underlying DR and its clinical manifestations, utilizing advancements in pharmacology, surgical techniques, and systemic management (Sun et al. 2023). Furthermore, a growing emphasis on personalized medicine, interdisciplinary management, and holistic patient-centered care underscores the evolving complexity of DR care. As the prevalence of diabetes continues to rise globally, understanding and addressing DR becomes increasingly imperative for preserving vision and improving quality of life (Yurievna and Victorovich 2022; Kamalu et al. 2024).

### Anti-VEGF therapy

The introduction of anti-VEGF agents represents a paradigm shift in the management of DR, particularly in DME and PDR. VEGF plays a central role in pathological angiogenesis and increased vascular permeability, making it a critical therapeutic target (Hsieh et al. 2022).

#### Mechanism of action

Anti-VEGF agents, including ranibizumab, aflibercept, and off-label bevacizumab, function by neutralizing VEGF-A isoforms. This inhibition reduces vascular permeability, retinal edema, and neovascularization, thereby stabilizing or improving visual acuity. VEGF blockade also mitigates inflammation and oxidative stress, providing ancillary benefits in disease stabilization. Novel agents targeting additional VEGF pathways are under investigation to further refine therapeutic outcomes (Srejovic et al. 2024).

#### Clinical application

These agents are delivered via intravitreal injections, with dosing regimens tailored to disease severity and activity. Common protocols include monthly injections, treat-and-extend schedules, or pro re nata (PRN) approaches (Nichani et al. 2023). Recent innovations in sustained-release formulations, such as port delivery systems and refillable implants, aim to reduce treatment burden while maintaining therapeutic efficacy. Investigational therapies incorporating bi-specific antibodies targeting VEGF and angiopoietin-2 offer new possibilities for more comprehensive vascular stabilization (Alshaikh et al. 2022; Iglicki et al. 2022).

#### Efficacy

Landmark trials, including DRCR.net Protocol T, have demonstrated the superior efficacy of anti-VEGF agents in improving visual outcomes compared to laser therapy or corticosteroids (Ehlers et al. 2022; Zhang et al. 2022a, b, c). Among these agents, aflibercept has shown particular efficacy in patients with severe visual impairment. Longitudinal data also suggest that early intervention with anti-VEGF therapy may prevent progression to advanced disease stages. Real-world studies increasingly validate these findings, though variations in outcomes highlight the importance of individualized care (Parravano et al. 2023).

While highly effective, these treatments are not without limitations. Frequent injections pose challenges related to patient adherence, financial costs, and the potential development of tachyphylaxis (Uludag et al. 2022). Furthermore, real-world studies indicate disparities in access to anti-VEGF therapy, necessitating policy interventions to ensure equitable care. Ongoing research into alternative delivery mechanisms, including ocular gene therapy, aims to address these challenges by offering potentially long-lasting therapeutic effects (Rowe and Ciulla 2024).

### Corticosteroid therapy

Corticosteroids represent a viable alternative or adjunctive treatment for patients with refractory DME or those unable to tolerate anti-VEGF therapy. Their anti-inflammatory properties target multiple pathways implicated in DR pathogenesis (Wilkins et al. 2022).

#### Mechanism of action

Corticosteroids suppress pro-inflammatory cytokines, reduce vascular permeability, and mitigate VEGF expression. Sustained-release implants, such as dexamethasone and fluocinolone acetonide, provide prolonged therapeutic effects. These implants utilize advanced polymer matrices to achieve consistent drug delivery over months or years, minimizing treatment burden (Xu et al. 2020; Baillif et al. 2022).

#### Clinical use

These implants are particularly beneficial for chronic DME and patients with a history of poor adherence to frequent injection regimens. They also demonstrate utility in combination therapies for refractory cases (Taloni et al. 2023). Emerging data suggest that corticosteroids may also have neuroprotective effects, which could extend their utility in earlier disease stages. Research exploring the integration of corticosteroid implants with advanced delivery technologies, such as biodegradable systems, is ongoing to further optimize outcomes (Tsung et al. 2023).

#### Limitations

Cataract progression and elevated intraocular pressure (IOP) are notable side effects that necessitate careful patient selection and monitoring. Ongoing research aims to refine corticosteroid formulations to minimize these adverse effects while enhancing efficacy (Lu et al. 2022). For example, novel bioerodible implants are under investigation to improve biocompatibility and safety profiles. Furthermore, combining corticosteroids with emerging systemic therapies may provide synergistic benefits for patients with complex disease profiles (Adán et al. 2020).

### Laser photocoagulation

While often replaced by pharmacologic therapies in numerous cases, laser photocoagulation retains a pivotal role in the treatment of DR, particularly in resource-limited settings or as an adjunct to other modalities (Wang et al. 2024a).

#### Panretinal photocoagulation

Panretinal photocoagulation (PRP) is the standard of care for PDR, reducing ischemic retinal areas to limit VEGF production (Zhang et al. 2022a, b, c). While effective in preventing severe vision loss, PRP is associated with adverse effects, including peripheral vision loss and diminished night vision (Pandya et al. 2024). Advances in laser platforms, such as multispot lasers and automated delivery systems, have reduced procedural duration and patient discomfort, improving overall safety and tolerability (Azarcon and Artiaga 2021).

#### Focal/grid laser therapy

This approach is utilized in select cases of DME, particularly in conjunction with pharmacologic interventions. Advances in laser technology, such as subthreshold micropulse lasers, have improved treatment precision, reducing collateral damage and enhancing safety profiles (Wang et al. 2024a). Such innovations have expanded the applicability of laser therapy, particularly in early or mild disease stages. Moreover, combining laser therapy with anti-VEGF or corticosteroid treatments has shown promise in achieving synergistic benefits (Chalke and Kale 2021).

### Surgical intervention

Surgical techniques, particularly pars plana vitrectomy, are reserved for advanced stages of DR complicated by vitreous hemorrhage or tractional retinal detachment (Barazani 2024).

#### Procedure and outcomes

Vitrectomy involves the removal of vitreous opacities and fibrous membranes, coupled with endolaser photocoagulation to address underlying ischemia (Patel et al. 2024). Recent advancements, including high-resolution intraoperative imaging and smaller-gauge instrumentation, have enhanced procedural outcomes. Moreover, the integration of intraoperative OCT has facilitated real-time visualization of retinal structures, improving surgical precision. These innovations have reduced recovery times and complication rates, broadening the accessibility of surgical interventions (Lai et al. 2021; Silpa-archa et al. 2024).

#### Adjunctive therapies

Preoperative anti-VEGF administration has been shown to reduce intraoperative bleeding and postoperative recurrence of neovascularization (Zhao et al. 2023). Emerging technologies, such as robotic-assisted surgery and novel vitreous substitutes, are poised to further refine surgical precision and safety. Additionally, postoperative use of sustained-release drug implants is being explored to prevent disease recurrence (Xu et al. 2023). Combination approaches, integrating surgery with systemic therapies, are gaining traction as comprehensive strategies for managing advanced disease stages (Tan et al. 2020).

### Systemic optimization

Optimal management of DR necessitates a comprehensive approach, addressing systemic risk factors that drive disease progression. Metabolic control remains foundational in this regard (Yumnamcha et al. 2020).

#### Glycemic control

Evidence from pivotal studies, including the Diabetes Control and Complications Trial (DCCT) and UK Prospective Diabetes Study (UKPDS), underscores the importance of tight glycemic control in reducing the incidence and progression of DR (Lachin and Nathan 2021). Modern tools, such as continuous glucose monitors (CGMs) and automated insulin delivery systems, enhance glycemic management. Long-term adherence to glycemic targets has also been associated with sustained benefits in DR prevention. Novel biomarkers are under investigation to enable earlier detection of glycemic excursions linked to DR risk (Everett 2024).

#### Adjunctive pharmacotherapies

Agents such as fenofibrate and angiotensin receptor blockers (ARBs) have demonstrated ancillary benefits in DR by modulating lipid metabolism and vascular homeostasis (Tang et al. 2024). Sodium-glucose co-transporter-2 (SGLT2) inhibitors are also being explored for their potential retinal protective effects (Lahoti et al. 2021). Emerging evidence suggests that anti-inflammatory and antioxidant agents may complement existing therapies, providing broader systemic benefits. Furthermore, tailored interventions targeting individual metabolic profiles are gaining prominence in optimizing outcomes (López-Contreras et al. 2020).

### Future directions in the management of DR

Emerging strategies for DR management emphasize early detection, novel therapeutic modalities, and personalized care paradigms aimed at enhancing patient outcomes and quality of life. These advancements are transforming the field of ophthalmology, paving the way for a future where vision loss caused by DR could be greatly reduced through comprehensive and integrated care systems (Pei et al. 2024).

### Advancements in early detection and screening

The early identification of DR is pivotal for preventing severe visual complications. Key advancements in this area include:

- Biomarkers for risk stratification: Ongoing investigations into systemic and ocular biomarkers, such as VEGF, AGEs, and inflammatory cytokines, are offering novel avenues for stratifying patients based on their risk of DR progression (Sun et al. 2024). These biomarkers provide a molecular framework for predicting disease trajectory and may eventually serve as therapeutic targets, facilitating personalized intervention strategies. Innovations in high-throughput screening and bioinformatics are accelerating the discovery of additional biomarkers that can further refine risk stratification (Zhang et al. 2024).

### Innovations in pharmacological interventions

While traditional therapies such as laser photocoagulation and anti-VEGF agents remain standard, novel pharmacological strategies are rapidly emerging:

- Next-generation anti-VEGF therapies: Innovations in drug delivery, such as extended-release formulations and gene therapy approaches targeting VEGF, are designed to alleviate treatment burden and enhance compliance (Diress et al. 2024). Devices like port-delivery systems and advancements in intraocular gene editing represent promising developments. These novel approaches aim to mitigate the frequency of intravitreal injections, a significant barrier to treatment adherence. Additional research is exploring the efficacy of combination therapies that target multiple angiogenic pathways simultaneously (Liu and Yu 2024).
- Neuroprotective therapies: Emerging pharmacological interventions aim to mitigate retinal neuronal degeneration in the early stages of DR. Agents such as brimonidine and ciliary neurotrophic factor (CNTF) are currently being explored in preclinical and clinical settings (Oshitari 2024). These neuroprotective agents represent a shift in focus from vascular to neuronal health, addressing a previously underexplored side of DR pathology. Future research is expected to investigate combination therapies that integrate neuroprotective and anti-angiogenic mechanisms for holistic disease management (Biswas et al. 2024; Ye et al. 2024).

### Regenerative medicine and tissue engineering

Regenerative approaches are transforming DR treatment paradigms by focusing on tissue restoration:

- Stem cell therapies: Retinal pigment epithelial (RPE) cells derived from induced pluripotent stem cells (iPSCs) hold promise for replacing damaged retinal cells. Early clinical trials have demonstrated safety, though challenges such as immune rejection and functional integration persist (Galgani et al. 2024). Research is also investigating methods to enhance survival, differentiation, and integration of transplanted cells into the retinal structure. Moreover, advances in bioengineering are enabling the creation of retinal organoids, which serve as in vitro models for testing new therapeutic approaches (de Lemos et al. 2024).
- Retinal implants and prostheses: Advances in bioengineered retinal prosthetics and microelectronic implants offer hope for vision restoration in advanced stages of DR. These technologies aim to interface with neural circuits to partially restore visual perception. Moreover, hybrid approaches that combine cellular and prosthetic elements are emerging as a promising avenue for addressing complex retinal damage (Lanzani et al. 2024; Rizwan and Kumar 2024).

### Personalized and precision medicine

Tailoring DR management based on individual genetic, molecular, and clinical profiles represents a paradigm shift in therapeutic approaches (Reddy et al. 2024; Rowe and Ciulla 2024):

- Genetic profiling: Understanding genetic susceptibilities to DR progression, such as polymorphisms in VEGF and inflammatory pathway genes, enables stratified treatment planning and optimization of therapeutic outcomes. Advances in genomics are also aiding in identifying novel drug targets and creating therapies that cater to specific genetic subpopulations (Pei et al. 2024). Genome-editing tools like CRISPR are being explored for their potential to directly modify pathogenic genetic variants associated with DR (Lotfi et al. 2024).
- Metabolomics and proteomics: Comprehensive analyses of metabolic and protein alterations are yielding individualized biomarkers to guide therapeutic decision-making and monitor treatment efficacy. Integration of omics data with clinical and imaging findings is poised to create predictive models that can revolutionize disease management (Amiri-Dashatan et al. 2024; Soares et al. 2024).
- Nanotechnology-driven drug delivery: Innovations in nanotechnology, including nanoparticles and microneedle systems, are enhancing the precision and bioavailability of drug delivery while reducing systemic side effects. These approaches also hold potential for combining multiple drugs into a single delivery platform, thereby simplifying treatment regimens. Ongoing research is exploring the use of biodegradable nanocarriers to ensure sustained release and minimize the need for repeat administrations (Parashar et al. 2024; Wu et al. 2024).

### Lifestyle and behavioral interventions

Holistic approaches to DR management are gaining recognition for their role in disease prevention and control:

- Digital health tools: Mobile health applications and wearable devices empower patients to actively monitor glucose levels, track lifestyle modifications, and adhere to prescribed treatment regimens. These tools are increasingly incorporating gamification and social support features to encourage sustained engagement (Liu and Yu 2024).
- Behavioral interventions: Innovative strategies based on behavioral economics are being implemented to improve adherence to follow-up care and therapeutic protocols, ultimately enhancing long-term outcomes (Madit et al. 2024; Mossburg et al. 2024). For example, financial incentives, nudges, and personalized reminders are being tested in pilot programs to increase treatment compliance. Integrating culturally tailored interventions could further improve their effectiveness in diverse populations (Sanders et al. 2024).

## Natural compounds/herbal medicine interacting with one or more targets and evidence supporting their use in the management of DR

By being more culturally accepted, more affordable, and generally tolerated with minimum side effects, herbal medicine continues to be the primary source of healthcare for approximately 75% of the world’s population particularly in underdeveloped and developing nations. Nonetheless, their application in developed world has significantly increased in recent years (Sam 2019). DR has been managed using a variety of plant constituents and herbal medicines (Kumar et al. 2021). They have been proven their effectiveness in treatment and prevention of DR either alone or in combination with conventional therapy (Ansari-Mohseni et al. 2023). Among the most popular herbal remedies was the Chinese herbal medicine which included the use of *Panax ginseng*, wolfberry, *Astragalus membranaceus*, *Ginkgo biloba*, and other traditional Chinese herbs to control DR (Behl and Kotwani 2017; Ai et al. 2020). As demonstrated by a considerable number of in vivo and in vitro studies, flavonoids as a class of plant constituents exhibited various beneficial effects in DR by preserving blood retinal barrier integrity, suppression of proinflammatory cytokines release, ameliorating retinal oxidative damage and apoptosis (Matos et al. 2020). The potential of numerous other natural compounds and herbal supplements in DR was evaluated by employing various targets and mechanisms (Table 1).

**Table 1:** Natural compounds/herbal medicine interacting with one or more targets in the management of diabetic retinopathy (DR)

Plant/herbal supplement	Part used	Active principle/herbal supplement composition	Chemical class	Type of study	Result	References
Wolfberry/Goji berry *Lycium barbarum*	Fruit	Lycium Barbarum Polysaccharides (LBP)	Carbohydrates	In vivo	Downregulation of proangiogenic factors VEGF and GFAP and inversely upregulating antiangiogenic factor PEDF Ameliorated diabetes-associated retinal structure alterations and enhanced overall retinal function	(Yao et al. 2018)
		Zeaxanthin and lutein	Carotenoids	In vivo	Retinal protective effect via AMPK-α2 activation in mitochondria and nuclei leading to upregulation of lutein and zeaxanthin metabolic genes SR-BI, GSTP1, and BCO2 Relieved mitochondrial stress and hypoxia by downregulating HIF-1α, VEGF, and HSP60, besides the improvement of mitochondrial biogenesis expressed by increased PGC-1α, NRF1, and TFAM	(Tang et al. 2011; Yu et al. 2013)
Saffron *Crocus sativus*	Trifid stigma Styles	Crocin Total extract	Carotenoid	Cell line	Alleviated DR-associated microglial activation triggered in the study by glucose and fatty acids treatment by virtue of its anti-inflammatory and antioxidant properties Neuroprotective benefits via PI3K/Akt pathway	(Yang et al. 2017)
					Anti-VEGF properties and functions to inhibit angiogenesis Downregulated MMP-2 and MMP-9 genes expression. Anti-inflammatory and angiogenesis-suppressing agents	(Sepahi et al. 2021)
				In vivo	Reduce lipid peroxidation and restore antioxidant protective enzymes activity in DR via reduction in levels of toxic MDA and elevation of GPx and SOD enzymes activities	(Skourtis et al. 2020)
Blueberry	Fruit	Anthocyanins	Flavonoids	In vivo	Enhancements in radicals scavenging capacity, amplification in retinal GSH content and the activity of GPx enzyme, in addition to attenuating MDA and retinal oxidative stress Anti-inflammatory actions of VEGF and IL-1β Modification in Nrf2/HO-1 signaling pathway	(Song et al. 2016)
				In vitro	Attenuation in oxidative stress, endoplasmic reticulum stress and apoptosis associated with DR in ARPE-19 cells besides downregulating miR-182 which directly inhibits OGG1	(Wang et al. 2022)
		Cyanidin-3-*O*-glucoside		In vivo	Anti-inflammatory and antioxidant actions by acting on REDD1 protein in the choroid-RPE of diabetic mice C3G in particular, regulated the redox balance in retinal cells through targeting REDD1 lowering VEGFA elevated in DR	(Li et al. 2024c)
Bilberry or whortleberry *vaccinium myrtillus L*	Fruit	Anthocyanins	Flavonoids	In vivo	Remarkable improvement in early-stage DR breakdown of blood retinal barrier through inhibiting VEGF primarily by delphinidin, as well as ameliorating loss of tight junction protein which progresses to macular edema in late-stage DR	(Kim et al. 2015)
				In vivo	Downregulation of the apoptosis-regulating proteins crystallins was observed in HFD with bilberries mice compared to HFD group, moreover it reduced gene expression in pathway of MAPK and increased gene expression in glutathione metabolism pathway exerting an anti-inflammatory, antioxidant and anti-apoptotic effects in DR	(Mykkänen et al. 2012)
Turmeric *Curcuma longa*	Rhizome	Curcumin	Flavonoids	Clinical	Inhibition in proinflammatory cytokines: TNF-α, IL2, and the angiogenic factor PDGF-AB alleviating retinal injury in DR	(Kocaadam and Şanlier 2017; Filippelli et al. 2021)
				In vivo	Altering structural changes in diabetic rat retina involving retinal thinning, retinal capillary basement membrane thickening, retinal ganglion and inner nuclear layer cell apoptosis, and photoreceptor cell membranous disks disruption. Furthermore, curcumin inhibited retinal VEGF and proapoptotic protein Bax, along with inducing antiapoptotic protein Bcl-2 expression prompting antioxidant and anti-apoptotic effects	(Yang et al. 2018)
				In vivo	Curcumin had a powerful apoptosis inhibitory effect compared to insulin as well as a considerable anti-edematous effect on diabetic retina. Antioxidant power of curcumin discouraged the Nrf2 pathway activated in early-stage DR to balance the increase in oxidative stress The effect on Nrf2 pathway was superior in rats cotreated with insulin and curcumin than that of insulin treated group	(Xie et al. 2021)
				Cell line	Antioxidant and anti-apoptotic activities of curcumin on high glucose RRVECs. It was found that curcumin significantly diminished the elevated NF-κB and phosphorylated NF-κB activity brought on by high glucose concentration in RRVECs confirming its protective effect in DR	(Huang et al. 2021a)
*Astragalus membranaceus* (Huang qi) (Chinese herbal medicine)	Root	Quercetin, kaempferol, and astragaloside IV	Flavonoids	Network pharmacology study	Astragaloside IV, kaempferol, and quercetin were among the 24 active chemicals that were detected in AM After further screening of the 169 AM targets that were found to be efficacious in controlling DR, 38 core targets including VEGFA, AKT1, and IL-6 genes were ultimately identified Antioxidant, anti-apoptotic, anti-inflammatory, and anti-angiogenic effects were achieved via targeting many metabolic pathways, for instance, PI3K-Akt signaling system, EGFR tyrosine kinase inhibitor resistance and AGE-RAGE signaling axis in diabetic complications	(Fu et al. 2014; Jin et al. 2020)
*Ginkgo biloba* L (Maiden hair tree)	Leaf	Quercetin, kaempferol, iso-rhamnetin, myricetin, laricitrin, mearnsetin, and apigenin glycosides -Ginkgetin and isoginkgetin -Ginkgolides A, B, C, and J -Bilobalide	-Flavonoids	Clinical	Administration of ginkgo biloba extract by DR patients significantly improved retinal capillary blood flow rate	(Huang et al. 2004)
			-Biflavonoids -Diterpene lactones -Sesquiterpene lactone	Cell lines	*Ginkgo biloba* extract demonstrated antioxidant activity via upregulating AMPK, Nrf2, and NQO-1 antioxidant pathway thus preserving human retinal Müller glial cell	(Li et al. 2023)
Fortified Extract of Red berry, *Ginkgo biloba*, and White Willow Bark	-	α-lipoic acid, Salix alba extract (15% salicin), berry extract (35% polyphenols; 6% anthocyanins), *Ginkgo biloba* extract (22.0–27.0% Ginkgo flavonoids; 5.0–7.0% terpene lactones; ginkgolic acid content < 5 ppm), and L-carnosine In recommended doses	Flavonoids	In vivo	Retinal protective effects of fortified extract in early-stage diabetic rats were manifested through its anti-inflammatory action by means of reducing TNF-α and VEGF cytokines Treatment with the fortified extract significantly lowered retinal cytokine levels and suppressed diabetes-related lipid peroxidation. These data demonstrate that the fortified extract attenuates the degree of retinal inflammation and plasma lipid peroxidation preserving the retina in early diabetic rats	(Bucolo et al. 2013)
Pills of six ingredients with rehmannia combined with *Ginkgo biloba* leaf	-	-	Flavonoids	Clinical	Study groups presented with delayed rate of progression and increased rate of remission of DR compared to control without any significant adverse effect	(Yang 2017)
North *American ginseng*	Root	Ginsenosides Rg1, Re, Rb1, Rc, Rb2, and Rd. mainly Rb1 and Re	Saponins	In vivo	Ginseng considerably relieved retinal oxidative stress by upregulating the powerful antioxidant enzyme superoxide dismutase, additionally ginseng attenuated extracellular matrix proteins: fibronectin and its extradomain B positive splice variant (EDB + FN), and vasoactive factors (VEGF and endothelin 1) elevated in DR	(Sen et al. 2013)
*Panax ginseng*	Root	Ginsenoside Rg3	Saponin	In vivo	Ginsenoside Rg3-treated group presented with an inhibition of diabetes-induced neovascularization and downregulation of VEGF and TNF-α as compared to diabetic control. Compared to normal control, ginsenoside Rg3 alone was not effective enough to protect from DR as the levels of VEGF and TNF-α were higher in treated group than normal negative control group	(Sun and Zhou 2010)
		Ginsenoside Rb1	Saponin	Cell line	Ginsenoside Rb1 diminished the production of VEGF by retinal epithelial cells suppressing angiogenesis and indicating its potential in DR prevention	(Betts et al. 2011)
		Ginsenoside Rk1	Saponin	Cell line	The study revealed that Ginsenoside Rk1 had a positive impact on diabetes-induced blood retinal barrier breakdown by inhibiting VEGF which is a major cause of increased retinal blood vessel permeability and consequent edema in DR	(Maeng et al. 2013)
		Ginsenosides Rg1, Re, Rh1, and Rd (crude saponins of Panax notoginseng (CSPN))	Saponins	Cell line	Retinal neuron loss due to apoptosis in early DR was ameliorated by CSPN in addition to postsynaptic protein PSD-95 loss, furthermore CSPN exhibited an antioxidant activity relieving endoplasmic reticulum stress	(Wang et al. 2016)
Fenugreek *Trigonella foenum-graecum* Linn	Seeds	Diosgenin	Saponins	In vivo	Fenugreek-treated rats demonstrated a decrease in DR associated elevation of angiogenic markers VEGF and PKC-β, and consequent protection against blood retinal barrier breakdown and neovascularization. Inflammatory cytokines were also inhibited by fenugreek particularly TNF-α and IL-1β. Additionally, diabetic rats given fenugreek displayed augmented retinal antioxidant enzymes (SOD and CAT) activity and GSH levels	(Gupta et al. 2014; Mishra 2024)
				In vivo	In comparison to the diabetic control group, diosgenin-treated mice showed a significant improvement in total retinal thickness, photoreceptor and outer nuclear layer thickness, and ganglion cell loss along with apoptosis suppression	(Liao et al. 2023)
Garlic (*Allium sativum*)		Allyl propyl disulfide, allicin, cysteine sulfoxide, and S-allyl cysteine sulfoxide	Sulfur-containing compounds	In vivo	The study demonstrated that garlic extract delayed DR progression as it slowed down the deterioration of retinal neuronal cells and layers by restoring normal retinal thickness, cell diameter, and number of ganglion and photoreceptor cells. Likewise, garlic extract protected retinal vasculature in diabetic study group indicating the potential of garlic as a dietary supplement in DR control	(Bastaki et al. 2024)
				In vivo	Garlic treated rats displayed a reduction in oxidative stress markers relative to untreated diabetic group, implying the antioxidant properties of garlic. Additionally, TGF-β2 and IL-1β were downregulated by garlic exposing the anti-inflammatory activity of garlic extract and its potential in DR treatment	(Ziamajidi et al. 2022)
		Allicin	Sulfur-containing compound	In vivo	In a rat model of type 2 diabetes, the study showed that allicin therapy effectively mitigated inflammation, oxidative stress-induced pyroptosis, metabolic anomalies, and retinal histopathological changes. Furthermore, the study clarified the key mechanisms by which Allicin provides retinal protection, emphasizing its capacity to inhibit NLRP3 inflammasome-mediated pyroptosis and stimulate PINK1-parkin-mediated mitophagy in diabetic retina	(Xu and Yu 2024)
Garlic tablets	-	-	-	Clinical study	Strengthening visual acuity and lowering IOP. However, CMT was not significantly affected by garlic. Revealing that garlic tablets can possibly serve as an adjuvant therapy in management of DR. Diabetic patients tolerated garlic well, and there were no notable side effects that would have disrupted the safety profile	(Afarid et al. 2022)

*AMPK*, AMP-activated protein kinase; *SR-BI*, scavenger receptor class B type I; *GSTP1*, glutathione S-transferase Pi 1; *BCO2*, β,β-carotene 9′,10′-oxygenase 2; *HIF-1α*, hypoxia-inducible factor 1α; *VEGF*, vascular endothelial growth factor; *HSP60*, heat shock protein 60; *PGC-1α*, peroxisome proliferator-activated receptor γ co-activator-1α; *NRF1*, nuclear respiratory factor 1; *TFAM*, transcription factor A, mitochondrial; *STZ*, streptozotocin; *DR*, diabetic retinopathy; *GFAP*, glial fibrillary acid protein; *PEDF*, pigment epithelium derived factor; *BA*, blueberry anthocyanins; *GSH*, glutathione; *GPx*, glutathione peroxidase; *MDA*, malondialdehyde; *IL-1β*, interleukin-1beta; *Nrf2*, nuclear factor erythroid-derived 2-like 2; *HO-1*, heme oxygenase-1; *BAE*, blueberry anthocyanin extract; *OGG1*, 8-oxoguanine-DNA glycosylate; *C3G*, cyanidin-3-O-glucoside; *REDD1*, regulated in development and DNA damage 1; *choroid-RPE*, choroid-retinal pigment epithelium; *VEGFA*, vascular endothelial growth factor A; *MAPK*, mitogen-activated protein kinase; *t-BHP*, tert-butyl hydroperoxide; *AMPK*, AMP-activated protein kinase; *NQO-1*, NADPH quinone oxidoreductase 1; *TNF-α*, tumor necrosis factor-alpha; *AGE*, advanced glycation end products; *NF-κB*, nuclear factor kappa B; *AKT1*, gene coding for RAC-alpha serine/threonine-protein kinase; *IL6*, interleukin-6; *PI3K*, phosphoinositide 3-kinases; *EGFR*, epidermal growth factor receptor; *RAGE*, receptor of advanced glycation end products; *TGF-β2*, transforming growth factor beta-2; *CMT*, central macular thickness; *IOP*, intra-ocular pressure; *MMP-2*, matrix metalloproteinases 2; *MMP-9*, matrix metalloproteinases 9; *SOD*, superoxide dismutase; *CAT*, catalase enzyme

### Zeaxanthin and lutein

Two major carotenoids from wolfberry that are primarily concentrated in retinal tissue (Kowluru et al. 2008) have proven their potential in DR as experimented in type-2 diabetic mice. Results demonstrated their ability to protect the retina against apoptosis and aid in retinal structure reestablishment. The suppression of retinal apoptosis is owed to the regulation of gene expression via AMPK activation (Yu et al. 2013), which in turn upregulates retinal FOXO3α leading to an increase in expression of thioredoxin and Mn superoxide dismutase (Mn SOD), powerful antioxidant enzymes, relieving cellular oxidative stress (Kowluru et al. 2008; Tang et al. 2011). Activation of AMPK also increases the expression of a set of metabolic genes denoted as SR-BI, GSTP1, and BCO2 which corrects the hyperglycemia-induced modulation of zeaxanthin and lutein homeostasis in addition to genes responsible for mitochondrial biogenesis (PGC-1-α, NRF1, and TFAM), and cell stress responses (HIF-1, VEGF, and HSP60) eventually improving mitochondrial dysfunction caused by hyperglycemia and exerting a retino-protective effect on diabetic mice (Yu et al. 2013). Zeaxanthin has also shown a considerable inhibition of diabetes-induced elevation of VEGF (Kowluru et al. 2008).

### *Lycium barbarum* polysaccharides (LBP) 

LBP from wolfberry are widely known for their neuroprotective effect, a study conducted on mice affirmed this effect in retinal neurons, exposing the capability of LBP to conserve retinal neurons and prevent or slow down the progression of DR (Kwok et al. 2019). Furthermore, LBP was reported in another study on diabetic rats to maintain retinal function and integrity by means of restoring proangiogenic and antiangiogenic factors balance, minimizing neovascularization (Yao et al. 2018).

### Bilberry

The extract of bilberry is composed of 15 distinct anthocyanins (Nakajima et al. 2004). An in vivo study on streptozotocin (STZ)-induced diabetic rats showed that bilberry extract given at a dose of 100 mg/kg prevented the early-stage diabetes-induced blood retinal barrier breakdown by downregulating VGEF most potently by delphinidin anthocyanidine and reversed the tight junction protein loss occurring in DR, protecting from progressive macular edema and vision loss in late-stage DR (Kim et al. 2015). Elevated gene expression of crystallins (an apoptosis regulating proteins in mice) was observed in animal models of retinal diseases including DR; in a study, this was induced by a high fat diet in mice; compared to a group of high fat diet consuming bilberries, bilberry downregulated crystallins in addition to decreased gene expression in pathway of mitogen-activated protein kinase (MAPK) and enhanced gene expression in the glutathione metabolism pathway eliciting an anti-inflammatory, antioxidant, and anti-apoptotic effects (Mykkänen et al. 2012). Previous studies on DR patients using bilberry fruit extract in doses ranging from 160 to 500 mg showed an improvement in retinal blood vessel permeability and overall retinal health (Vaneková and Rollinger 2022).

### *Ginkgo biloba* extract

Based on multiple studies of the effect of *Ginkgo biloba* extract on pathogenesis of DR, it was found to have positive impact through multiple mechanisms. Fundamentally reducing ROS induced by nitric oxide as well as suppressing its production and hence the rate of retinal apoptosis. The diterpene lactone, ginkgolide B, counteracts the damage occurring in endothelial cells of retina by downregulating platelet activation factor resulting in a decrease in retinal vasculature leucocyte adhesion that triggers an inflammatory response, thus protecting retina from inflammation and subsequent retinal detachment (Behl and Kotwani 2017). Additionally, *Ginkgo biloba* extract suppresses the increase in VEGF and TNF-α characteristic of DR. Besides the anti-inflammatory and blood supply enhancement activity that all support its use in DR, both terpenoid and flavonoid fractions of the extract exhibit neuroprotective and anti-apoptosis action. However, apart from other antioxidants, the flavonoid fraction has a remarkable ability to act at mitochondrial level (Martínez-Solís et al. 2019).

### *Panax ginseng*

Not mentioning the antidiabetic activity of ginseng root extract corresponding to its saponin content, *Panax ginseng* saponins have also participated in pathogenesis of DR by their hydroxyl and superoxide radical scavenging ability rendering them potential antioxidant and therefore anti-apoptotic agents. They also act as anti-inflammatory agents via inhibiting monocyte chemoattractant protein-1 (MCP-1), NF-κB, TNF-α, and ILs, all are proinflammatory mediators involved in inflammatory downstream events occurring in DR. Along with the inflammation and oxidative stress inhibitory effect of the total root extract, the saponin ginsenoside Rb1 has particularly shown an anti-angiogenic activity through downregulation of the powerful pro-angiogenic factor: VEGF resulting in retinal protection against VEGF-induced proliferation and subsequent neovascularization associated with proliferative DR. A different saponin of the extract, ginsenoside Rk1, was also responsible for alleviating retinal edema by means of reducing permeability of retinal endothelium caused by VEGF and AGEs as well as maintaining endothelial barrier integrity by stabilizing tight junction proteins at the boundary of the barrier (Behl and Kotwani 2017).

### Curcumin

The polyphenolic compound curcumin from turmeric rhizome (Kocaadam and Şanlier 2017) has confirmed its effectiveness in managing DR through its antioxidant, anti-inflammatory, and anti-apoptotic activities (Rohilla et al. 2023). Curcumin restored the levels of retinal glutathione crucial in redox signaling process in animal models and decreased malondialdehyde (MDA) levels resulting in reduction of oxidative stress and downregulation of retinal NF-κB, meanwhile augmenting the activity of antioxidant enzyme superoxide dismutase and catalase and blocking cyclooxygenase (COX), lipoxygenase (LOX), and xanthine oxidase (XOD) enzyme activity. Moreover, curcumin has the ability to prevent the elevation of retinal nitrotyrosine levels contributing to retinal damage (Yang et al. 2021). The anti-inflammatory effect of curcumin is achieved by targeting peroxisome proliferator-activated receptor-γ (PPAR-γ), producing multiple retinal protective effects; first of all, it inhibits several inflammatory biomarkers, for instance, MCP-1, IL-6, matrix metalloproteinase-9 (MMP-9), C-reactive protein (CRP), etc., and preserve retinal barrier integrity. Additionally, by acting on PPAR-γ curcumin suppresses nitric oxide (NO) production and inflammatory cytokines resulting in inflammation and oxidative stress discouragement. Furthermore, curcumin significantly inhibits the inflammatory factor TNF-α resulting in anti-edematous effect. Lastly, curcumin’s anti-apoptotic activity is due to countering retinal elevated levels of VEGF (Aldebasi et al. 2013; Yang et al. 2021; Rohilla et al. 2023).

### *Astragalus membranaceus*

According to a considerable number of studies, *Astragalus membranaceus* had numerous beneficial effects in diabetic retinopathy. It enhanced visual acuity and fundus manifestations in diabetic patients, additionally reducing fasting blood glucose, triglycerides, plasma viscosity, and RBC aggravation index (Cheng et al. 2013). More importantly, the extract of *Astragalus membranaceus* was reported to possess anti-apoptosis along with anti-inflammatory activity by attenuating multiple inflammatory cytokines such as NF-κB, IL-1b, and TNF-α. It also decreases cellular proliferation and differentiation by ERK1/2 phosphorylation inhibition improving proliferative DR. Furthermore, it reverses the pathological outcomes resulting from upregulation of polyol pathway occurring in DR through inhibition of aldose reductase enzyme prompting ROS suppression and subsequent reduction of retinal apoptosis at the same time exerting an anti-inflammatory effect in retinal microglia. An anti-angiogenic action of the extract was also observed and was specifically contributing to Astragalin flavonoid that reduces VEGF, a popular target for management of DR (Behl and Kotwani 2017). Biochanin A, an isoflavanoid from *Astragalus membranaceus*, was also reported to exhibit protein glycosylation inhibitory effect preventing the formation of AGEs responsible for retinal damage in DR (Song et al. 2012).

### Fenugreek extract

Polyphenolics and tannins in fenugreek extract were able to ameliorate DR induced in rats, exerting their effect by lowering inflammatory cytokines IL-1β and TNF-α, as well as angiogenic factors VEGF and PKC-β defending retinal oxidative damage. Other various mechanisms are involved in the valuable effect of fenugreek extract in DR including recovery of retinal catalase and superoxide dismutase levels and vascular leakage suppression (Gupta et al. 2014; Mishra 2024). A natural steroidal sapogenin from fenugreek, diosgenin, was recently reported to possess an anti-apoptosis effect and therefore a possibility in diabetic retinopathy management (Liao et al. 2023), concluding that fenugreek extract exhibits anti-inflammatory, antioxidant, and anti-apoptotic activities in DR management.

### ent-9α-hydroxy-15-oxo-16-kauren-19-oic acid

A novel study revealed that the diterpenoid EKO has altered the vascular endothelium injury and oxidative stress attributed to DR both in vitro and in vivo. The mechanism established a new target in treatment of DR by upregulating ATXN3 protien/c-fos/focal adhesion signaling pathway, thus promoting endothelial integrity, and inhibiting cell apoptosis. Inhibition of cell apoptosis is also contributing to the antioxidant activity of EKO (Ge et al. 2024).

## Application methods for natural compounds/ herbal medicine in the management of DR

Herbal extracts are concentrated preparations of plants that have been utilized through years in traditional medicine. They are derived from several parts of the plant, including leaves, flowers, and roots, and contain potent chemicals that offer numerous health benefits (Shekar et al. 2015). These extracts can be prepared with a variety of solvents, including water, alcohol, glycerin, and vinegar, all of which absorb distinct phytochemicals from the plant (Kharbach et al. 2020).

Herbal extracts are available in a variety of forms, including liquid tinctures, powdered extracts, and essential oils. Liquid extracts are generally more convenient and absorbable than raw herbs. They are employed in a variety of applications, including oral pills and skin gels or lotions (Balekundri and Mannur 2020).

Meanwhile, they frequently confront challenges due to their low water solubility, which limits their bioavailability (Bommakanti et al. 2023). To address this, approaches such as nanotechnology-based formulations have been developed to increase solubility and provide sustained release (Chaudhary et al. 2023b). Another challenge is stability, as these extracts might degrade when exposed to light, heat, or oxygen (Gavanji et al. 2023). Also, advanced nanodelivery technologies including liposomes, nanoparticles, and microspheres can help preserve these extracts, increasing their stability and shelf life (Chaudhary et al. 2023a). The pharmacokinetics of herbal extracts, comprising absorption, distribution, metabolism, and elimination (ADME), are critical to their efficacy. Enhancing bioavailability, targeting ability, controlled release, and reduced toxicity via advanced carrier systems can greatly increase herbal bioactives’ absorption and therapeutic efficacy (Pawar et al. 2024).

Recent breakthroughs in nanotechnology have resulted in more effective delivery systems, with technologies such as nanoemulsions and phytosomes showing promise in improving the solubility, stability, and bioavailability of herbal therapies, hence increasing their effectiveness and reliability. Accordingly, several studies investigated the integration of nanotechnology-based approaches loaded with herbal medicines for the management of DR.

A previous study reported the potential of *Fructus Alpiniae zerumbet* loaded reactive oxidative species-responsive carrier system as a feasible antioxidant for prompt intervention in DR development (Li et al. 2024a). Another study demonstrated the efficiency of *Cyperus rotundus*-loaded zinc oxide nanoparticles when oral administered to DR-induced rats with resulted antidiabetic and anti-inflammatory effects against DR (Zhang et al. 2020).

Furthermore, a study postulated the efficacy of berberine loaded green synthesized gold nanoparticles in the management of diabetes and its microvascular complications such as DR owing to their ability to interfere with disease-causing proteins implicated in diabetic complications (Li et al. 2024b). Similarly, a further study demonstrated the potential of rutin-loaded gold nanoparticles in DR-induced rat models with enhanced retinal neural restoration, as well as inhibition in the opacity of eye lens (Moldovan et al. 2023).

Meanwhile, PLGA nanoparticles showed effectiveness in the topical delivery of pioglitazone to posterior eye segment for treatment of DR along with significant inhibition in the concentration of VEGF in vitreous fluid (Laddha and Kshirsagar 2020).

Moreover, a study showed the functionality of gelatin/poly glycerol sebacate nanoparticles injectable hydrogels loaded with sunitinib in the reduction of the production of VEGF and neovascularization in the retina relative to bevacizumab as a reference drug for DR (Pirmardvand Chegini et al. 2022). Furthermore, topical administration of hyaluronic acid grafted bovine serum albumin nanoparticles and loaded with apatinib proved their ability to selectively target CD44 positive retinal cells for treatment of DR, bypassing the harmful ocular side effects of the intravitreal route (Radwan et al. 2021).

In addition, the nanoformulation of quercetin in a zebrafish model of DR showed effectiveness in treatment of diabetic associated retinopathy compared to dexamethasone as a reference treatment (Wang et al. 2020). Additionally, it was previously established that the combination of injectable basal insulin and oral bioavailable curcumin-loaded double-headed PLGA nanoparticles significantly impeded the advancement of retinopathy and diabetic cataracts while also enhancing liver function and peripheral glucose homeostasis. Additionally, there was a contemporaneous decrease in lens aggregate protein and an increase in mitochondrial ATP synthesis (Ganugula et al. 2023).

Additionally, the activities of three crucial antioxidant enzymes superoxide, catalase, dismutase, and peroxidase present in the retinal tissues were also mimicked by ultrasmall Fe-Quer nanozymes created by coupling quercetin with low-toxic iron ions. As a result, these nanozymes demonstrated exceptional water dispersion and effective ROS scavenging capabilities in the treatment of DR. These nanoenzymes have been shown to have anti-inflammatory, anti-oxidative stress, anti-microvascular leakage, and anti-angiogenesis properties; in particular, they have been shown to have a protective effect on the blood vessels in early DR (Gui et al. 2023).

Concurrently, another study revealed that pigment epithelium-derived factor (PEDF) integrated in PLGA nanoparticles demonstrated the ability to lower ICAM-1 levels in retina cell cultures and hamper hypoxia-induced expression of VEGF. Furthermore, compared to PLGA-nanoparticles control, these nanoparticles significantly decreased retinal vascular inflammation and leakage in streptozotocin-induced DR rats for up to 5 weeks following intravitreal injection, and they significantly alleviated ischemia-induced retinal neovascularization in oxygen-induced DR rat model (Qu et al. 2022).

Another study investigated the employment of solid lipid nanoparticles ocuserts loaded with vildagliptin for sustained ocular delivery with outlined efficacy in attaining microvascular blood flow of the retina beyond blood glucose control, delineating them as a promising approach to treating DR (Elsayed et al. 2024). Besides, a smart targeted light-responsive polymeric microcapsule conjugated with avastin had presented successfulness on human retinal cells, which could potentially emerge as an effective tactic in the treatment of DR (Stoia et al. 2023).

Another attempt suggested that poloxamer hydrogel coupled with chitosan-N-acetyl-L-cysteine nanoparticles could improve loading and extend intravitreal delivery of avastin macromolecule in comparison to the free extract. This would reduce the frequency, risk, and cost of heavy intravitreal injections and improve treatment of posterior eye segment neovascularization and associated vitreoretinal disorders including DR (Taheri et al. 2022).

Additionally, curcumin nanoparticles grafted with chitosan derivatives and conjugated with deoxycholic acid showed efficacy to release curcumin continuously in a sustained manner as compared to free curcumin, thus inhibiting in vitro human retinal pigment epithelial (hRPE) cells and reducing the expression of VEGF mRNA in hRPE cells (Zheng et al. 2022). Also, gold nanoparticles loaded with *Tagetes erecta* L. (Marigold) proved their potency to suppress hyperglycemia-induced senescence in retinal pigment epithelium through the inhibition of lipid peroxidation compared to the free extract, suggesting their effectiveness in the management of DR (Park et al. 2023).

## Conclusions

The intricate nature of the various mechanisms contributing to the pathological changes in DR presents significant challenges in achieving effective treatment outcomes with existing therapies. Natural products represent a non-invasive therapeutic option with considerable potential and are likely to be utilized either independently or in conjunction with conventional Western treatments for the prevention of DR. Worthy, natural products have shown promising anti-inflammatory, antioxidant, antiangiogenic, and neuroprotective properties in several diabetic models. An increase in both preclinical and clinical studies was recommended to further confirm the efficacy of natural products in managing DR.

## Limitations and future directions

There is insufficient clinical evidence to support the effectiveness of natural remedies alone in treatment of DR. Clinical studies conducted on natural products only prove their efficacy in certain pathway in disease progression and it is not clear whether they are used to prevent or alleviate DR (Rossino and Casini 2019). Poor bioavailability of herbal drugs is another barrier to their use in disease treatment resulting from their limited solubility, poor absorption, fast metabolism, and clearance from the body (Rossino and Casini 2019; Atanasov et al. 2021). Formulating natural products using novel drug delivery systems such as nanoparticles, liposomes, or co-administration with bioenhancers may optimize the bioavailability of natural drugs (Chaudhary et al. 2023a, b). Natural products may also demonstrate various drug interactions especially with synthetic drugs producing undesirable effects. For instance, the risk of bleeding increases when ginseng, garlic, or *Ginkgo biloba*, proposed for alleviating DR, are combined with antithrombotic medications like aspirin or warfarin (Xie and Wang 2023).

Future research on natural products for DR should top the list in overcoming bioavailability issues to achieve therapeutic advances, where high-performance delivery systems—i.e., nanoparticles, liposomes, and sustained-release formulations—are promising to produce a new natural compound that has better stability, solubility, and absorption properties. Through the maintenance of sufficient pharmacological concentrations at intended sites, these technologies overcome a major bottleneck in the translation of natural products to the clinic. Additionally, the integration of bio enhancer agents that improve the pharmacokinetics of natural compounds may further optimize their efficacy in addition to conducting comprehensive safety evaluations to ensure minimal side effects. Performing large-scale, rigorous clinical trials to establish effectiveness in human populations and investigating synergistic effects with existing pharmacological treatments to maximize therapeutic outcomes are needed in future investigations (Zhao et al. 2024).

## Data Availability

All source data for this work (or generated in this study) are available upon reasonable request.

## References

[CR1] Abdelrahman AA, Powell FL, Jadeja RN, Jones MA, Thounaojam MC, Bartoli M, Al-Shabrawey M, Martin PM (2022) Expression and activation of the ketone body receptor HCAR2/GPR109A promotes preservation of retinal endothelial cell barrier function. Exp Eye Res 221:109129 35649469 10.1016/j.exer.2022.109129 PMC13215273

[CR2] Adán A, Cabrera F, Figueroa MS, Cervera E, Ascaso FJ, Udaondo P, Abraldes M, Reyes MÁ, Pazos M, Pessoa B (2020) Clinical-decision criteria to identify recurrent diabetic macular edema patients suitable for fluocinolone acetonide implant therapy (ILUVIEN®) and follow-up considerations/recommendations. Clin Ophthalmol 14:2091–2107 32801618 10.2147/OPTH.S252359 PMC7398681

[CR3] Afarid M, Sadeghi E, Johari M, Namvar E, Sanie-Jahromi F (2022) Evaluation of the effect of garlic tablet as a complementary treatment for patients with diabetic retinopathy. J Diabetes Res 2022:6620661 35875346 10.1155/2022/6620661 PMC9303161

[CR4] Ahuja S, Saxena S, Akduman L, Meyer CH, Kruzliak P, Khanna VK, Ahuja S, Saxena S, Akduman L, Meyer CH, Kruzliak P, Khanna VK (2019) Serum vascular endothelial growth factor is a biomolecular biomarker of severity of diabetic retinopathy. Int J Retina Vitreous 5:1–6 31583119 10.1186/s40942-019-0179-6 PMC6771093

[CR5] Ai X, Yu P, Hou Y, Song X, Luo J, Li N, Lai X, Wang X, Meng X (2020) A review of traditional Chinese medicine on treatment of diabetic retinopathy and involved mechanisms. Biomed Pharmacother 132:110852 33065390 10.1016/j.biopha.2020.110852

[CR6] Al Zabadi H, Taha I, Zagha R (2022) Clinical and molecular characteristics of diabetic retinopathy and its severity complications among diabetic patients: a multicenter cross-sectional study. J Clin Med 11:3945 35887709 10.3390/jcm11143945 PMC9319242

[CR7] Aldebasi YH, Aly SM, Rahmani AH (2013) Therapeutic implications of curcumin in the prevention of diabetic retinopathy via modulation of anti-oxidant activity and genetic pathways. Int J Physiol Pathophysiol Pharmacol 5:194 24379904 PMC3867697

[CR8] Aldosari DI, Malik A, Alhomida AS, Ola MS (2022) Implications of diabetes-induced altered metabolites on retinal neurodegeneration. Front Neurosci 16:938029 35911994 10.3389/fnins.2022.938029 PMC9328693

[CR9] Alshaikh RA, Waeber C, Ryan KB (2022) Polymer based sustained drug delivery to the ocular posterior segment: barriers and future opportunities for the treatment of neovascular pathologies. Adv Drug Deliv Rev 187:114342 35569559 10.1016/j.addr.2022.114342

[CR10] Amiri-Dashatan N, Etemadi SM, Besharati S, Farahani M, Moghaddam AK (2024) Dysregulation of amino acids balance as potential serum-metabolite biomarkers for diagnosis and prognosis of diabetic retinopathy: a metabolomics study. J Diabetes Metab Disord 23(2):2031–2042. 10.1007/s40200-024-01462-y 10.1007/s40200-024-01462-y PMC11599686 39610496

[CR11] Andrés-Blasco I, Gallego-Martínez A, Machado X, Cruz-Espinosa J, Lauro SD, Casaroli-Marano R, Alegre-Ituarte V, Arévalo JF, Pinazo-Durán MD, Andrés-Blasco I, Gallego-Martínez A, Machado X, Cruz-Espinosa J, Di Lauro S, Casaroli-Marano R, Alegre-Ituarte V, Arévalo JF, Pinazo-Durán MD (2023) Oxidative stress, inflammatory, angiogenic, and apoptotic molecules in proliferative diabetic retinopathy and diabetic macular edema patients. Int J Mol Sci 24:8227 10.3390/ijms24098227 PMC10179600 37175931

[CR12] Ansari-Mohseni N, Ghorani-Azam A, Mohajeri SA (2023) Therapeutic effects of herbal medicines in different types of retinopathies: a systematic review. Avicenna J Phytomed 13:118 37333471 10.22038/AJP.2022.62423.2977 PMC10274316

[CR13] Antonetti DA, Silva PS, Stitt AW (2021) Current understanding of the molecular and cellular pathology of diabetic retinopathy. Nat Rev Endocrinol 17:195–206 33469209 10.1038/s41574-020-00451-4 PMC9053333

[CR14] Atanasov AG, Zotchev SB, Dirsch VM, Supuran CT (2021) Natural products in drug discovery: advances and opportunities. Nat Rev Drug Discov 20:200–216 33510482 10.1038/s41573-020-00114-z PMC7841765

[CR15] Azarcon CP, Artiaga JCM (2021) Comparison of pain scores among patients undergoing conventional and novel panretinal photocoagulation for diabetic retinopathy: a systematic review. Clin Ophthalmol 15:953–971 33688163 10.2147/OPTH.S294227 PMC7936685

[CR16] Baillif S, Staccini P, Weber M, Delyfer M-N, Le Mer Y, Gualino V, Collot L, Merite P-Y, Creuzot-Garcher C, Kodjikian L (2022) Management of patients with diabetic macular edema switched from dexamethasone intravitreal implant to fluocinolone acetonide intravitreal implant. Pharmaceutics 14:2391 36365209 10.3390/pharmaceutics14112391 PMC9693281

[CR17] Balekundri A, Mannur V (2020) Quality control of the traditional herbs and herbal products: a review. Futur J Pharm Sci 6:1–9

[CR18] Bany Bakar R, Reimann F, Gribble FM (2023) The intestine as an endocrine organ and the role of gut hormones in metabolic regulation. Nat Rev Gastroenterol Hepatol 20:784–796 37626258 10.1038/s41575-023-00830-y

[CR19] Barazani H (2024) Risk factors of postoperative vitreous hemorrhage after pars plana vitrectomy for proliferative diabetic retinopathy (Master’s thesis, Lithuanian University of Health Sciences (Lithuania)

[CR20] Bastaki NK, Barhoush SA, Al-Adsani AM (2024) Garlic extract (Allium sativum L.) attenuates neurodegeneration and microvascular damage in rats with streptozotocin-induced diabetic retinopathy. J Funct Foods 122:106486

[CR21] Behl T, Kotwani A (2017) Chinese herbal drugs for the treatment of diabetic retinopathy. J Pharm Pharmacol 69:223–235 28124440 10.1111/jphp.12683

[CR22] Betts BS, Parvathaneni K, Yendluri BB, Grigsby J, Tsin AT (2011) Ginsenoside-Rb1 induces ARPE-19 proliferation and reduces VEGF release. Int Sch Res Notices 2011:184295 10.5402/2011/184295 PMC3912597 24527228

[CR23] Biswas A, Choudhury AD, Agrawal S, Bisen AC, Sanap SN, Verma SK, Kumar M, Mishra A, Kumar S, Chauhan M (2024) Recent insights into the etiopathogenesis of diabetic retinopathy and its management. J Ocular Pharmacol Ther 40:13–33 10.1089/jop.2023.0068 37733327

[CR24] Bommakanti V, Puthenparambil Ajikumar A, Sivi CM, Prakash G, Mundanat AS, Ahmad F, Haque S, Prieto MA, Rana SS (2023) An overview of herbal nutraceuticals, their extraction, formulation, therapeutic effects and potential toxicity. Separations 10:177

[CR25] Boscia G, Bacherini D, Vujosevic S, Grassi MO, Borrelli E, Giancipoli E, Landini L, Pignataro M, Alessio G, Boscia F, Viggiano P (2024) Long-term impact of diabetic retinopathy on response to anti-VEGF treatment in neovascular AMD. Invest Ophthalmol vis Sci 65:6–6 39093297 10.1167/iovs.65.10.6 PMC11305436

[CR26] Bryl A, Mrugacz M, Falkowski M, Zorena K (2022) The effect of diet and lifestyle on the course of diabetic retinopathy—a review of the literature. Nutrients 14:1252 35334909 10.3390/nu14061252 PMC8955064

[CR27] Bucolo C, Marrazzo G, Platania CBM, Drago F, Leggio GM, Salomone S (2013) Fortified extract of red berry, Ginkgo biloba, and white willow bark in experimental early diabetic retinopathy. J Diabetes Res 2013:432695 23762874 10.1155/2013/432695 PMC3676923

[CR28] Chalise U, Hale TM (2024) Fibroblasts under pressure: cardiac fibroblast responses to hypertension and antihypertensive therapies. Am J Physiol-Heart Circ Physiol 326:H223–H237 37999643 10.1152/ajpheart.00401.2023 PMC11219059

[CR29] Chalke SD, Kale PP (2021) Combinational approaches targeting neurodegeneration, oxidative stress, and inflammation in the treatment of diabetic retinopathy. Curr Drug Targets 22:1810–1824 33745432 10.2174/1389450122666210319113136

[CR30] Chaudhary D, Deep A, Bansal N, Rani N (2023a) Methods of synthesis, characterization and anticancer potential of herbal silver nanoparticles: a review. Curr Cancer Ther Rev 19:318–333

[CR31] Chaudhary J, Tailor G, Yadav M, Mehta C (2023b) Green route synthesis of metallic nanoparticles using various herbal extracts: a review. Biocatal Agric Biotechnol 50:102692

[CR32] Chen Y, Coorey NJ, Zhang M, Zeng S, Madigan MC, Zhang X, Gillies MC, Zhu L, Zhang T (2022) Metabolism dysregulation in retinal diseases and related therapies. Antioxidants 11:942 35624805 10.3390/antiox11050942 PMC9137684

[CR33] Cheng L, Zhang G, Zhou Y, Lu X, Zhang F, Ye H, Duan J (2013) Systematic review and meta-analysis of 16 randomized clinical trials of Radix Astragali and its prescriptions for diabetic retinopathy. Evid-Based Complement Alternat Med 2013:762783 23573153 10.1155/2013/762783 PMC3618930

[CR34] Dănilă A-I, Ghenciu LA, Stoicescu ER, Bolintineanu SL, Iacob R, Săndesc M-A, Faur AC, Dănilă A-I, Ghenciu LA, Stoicescu ER, Bolintineanu SL, Iacob R, Săndesc M-A, Faur AC (2024) Aldose reductase as a key target in the prevention and treatment of diabetic retinopathy: a comprehensive review. Biomedicines 12:747 38672103 10.3390/biomedicines12040747 PMC11047946

[CR35] de Lemos L, Antas P, Ferreira IS, Santos IP, Felgueiras B, Gomes CM, Brito C, Seabra MC, Tenreiro S (2024) Modelling neurodegeneration and inflammation in early diabetic retinopathy using 3D human retinal organoids. In Vitro Models 3:33–48 39872068 10.1007/s44164-024-00068-1 PMC11756505

[CR36] Diress M, Wagle SR, Lim P, Foster T, Kovacevic B, Ionescu CM, Mooranian A, Al-Salami H (2024) Advanced drug delivery strategies for diabetic retinopathy: a comprehensive review on current medications, delivery methods, device innovations, overcoming barriers, and experimental models. Expert Opin Drug Deliv 21:1859–1877 39557623 10.1080/17425247.2024.2431577

[CR37] Ehlers JP, Yeh S, Maguire MG, Smith JR, Mruthyunjaya P, Jain N, Kim LA, Weng CY, Flaxel CJ, Schoenberger SD (2022) Intravitreal pharmacotherapies for diabetic macular edema: a report by the American Academy of Ophthalmology. Ophthalmology 129:88–99 34446301 10.1016/j.ophtha.2021.07.009

[CR38] Elsayed MM, Elsayed A, Fouad MA, Mohamed MS, Lee S, Mahmoud RA, Sabry SA, Ghoneim MM, Hassan AH, Abd Elkarim RA (2024) Development and optimization of vildagliptin solid lipid nanoparticles loaded ocuserts for controlled ocular delivery: a promising approach towards treating diabetic retinopathy. Int J Pharm X 7:100232 38357578 10.1016/j.ijpx.2024.100232 PMC10864762

[CR39] Eshaq RS, Aldalati AMZ, Alexander JS, Harris NR (2017) Diabetic retinopathy: breaking the barrier. Pathophysiology 24:229–241 28732591 10.1016/j.pathophys.2017.07.001 PMC5711541

[CR40] Everett EM (2024) Leveraging continuous glucose monitors to reduce the risk of diabetic retinopathy. JAMA Netw Open 7:e240718–e240718 38446485 10.1001/jamanetworkopen.2024.0718

[CR41] Fenner BJ, Wong RL, Lam W-C, Tan GS, Cheung GC (2018) Advances in retinal imaging and applications in diabetic retinopathy screening: a review. Ophthalmol Ther 7:333–346 30415454 10.1007/s40123-018-0153-7 PMC6258577

[CR42] Ferrat LA, Vehik K, Sharp SA, Lernmark Å, Rewers MJ, She J-X, Ziegler A-G, Toppari J, Akolkar B, Krischer JP (2020) A combined risk score enhances prediction of type 1 diabetes among susceptible children. Nat Med 26:1247–1255 32770166 10.1038/s41591-020-0930-4 PMC7556983

[CR43] Filippelli M, Campagna G, Vito P, Zotti T, Ventre L, Rinaldi M, Bartollino S, Dell’Omo R, Costagliola C (2021) Anti-inflammatory effect of curcumin, homotaurine, and vitamin D3 on human vitreous in patients with diabetic retinopathy. Front Neurol 11:592274 33633656 10.3389/fneur.2020.592274 PMC7901953

[CR44] Flaxel CJ, Adelman RA, Bailey ST, Fawzi A, Lim JI, Vemulakonda GA, Ying G-s (2020) Diabetic retinopathy preferred practice pattern®. Ophthalmology 127:P66–P145 31757498 10.1016/j.ophtha.2019.09.025

[CR45] Flores MD, del Carmen Cortés Ginez M, Gutman LAB (2023) Biochemical mechanisms of vascular complications in diabetes. The Diabetes Textbook, pp 695–707

[CR46] Fu J, Wang Z, Huang L, Zheng S, Wang D, Chen S, Zhang H, Yang S (2014) Review of the botanical characteristics, phytochemistry, and pharmacology of Astragalus membranaceus (Huangqi). Phytother Res 28:1275–1283 25087616 10.1002/ptr.5188

[CR47] Galgani G, Bray G, Martelli A, Calderone V, Citi V (2024) In vitro models of diabetes: focus on diabetic retinopathy. J Cells 13:1864 10.3390/cells13221864 PMC11592768 39594613

[CR48] Gáll T, Pethő D, Erdélyi K, Egri V, Balla JG, Nagy A, Póliska S, Gram M, Gábriel R, Nagy P (2024) Heme: a link between hemorrhage and retinopathy of prematurity progression. Redox Biol 76:103316 39260060 10.1016/j.redox.2024.103316 PMC11415884

[CR49] Gallego PH, Craig ME, Hing S, Donaghue KC (2008) Role of blood pressure in development of early retinopathy in adolescents with type 1 diabetes: prospective cohort study. BMJ 337:a918. 10.1136/bmj.a918 18728082 10.1136/bmj.a918 PMC2526183

[CR50] Gangwani RA, Lian J, McGhee S, Wong D, Li KK (2016) Diabetic retinopathy screening: global and local perspective. Hong Kong Med J 22:486–495 27562988 10.12809/hkmj164844

[CR51] Ganugula R, Arora M, Dwivedi S, Chandrashekar D, Varambally S, Scott E, Kumar MR (2023) Systemic anti-inflammatory therapy aided by curcumin-laden double-headed nanoparticles combined with injectable long-acting insulin in a rodent model of diabetes eye disease. ACS Nano 17:6857–6874 36951721 10.1021/acsnano.3c00535

[CR52] Gao J, Tao L, Jiang Z (2023) Alleviate oxidative stress in diabetic retinopathy: antioxidant therapeutic strategies. Redox Rep 28:2272386 38041593 10.1080/13510002.2023.2272386 PMC11001280

[CR53] Garvin AM, De Both MD, Talboom JS, Lindsey ML, Huentelman MJ, Hale TM (2021) Transient ACE (angiotensin-converting enzyme) inhibition suppresses future fibrogenic capacity and heterogeneity of cardiac fibroblast subpopulations. Hypertension 77:904–918 33486989 10.1161/HYPERTENSIONAHA.120.16352 PMC7878436

[CR54] Gavanji S, Bakhtari A, Famurewa AC, Othman EM (2023) Cytotoxic activity of herbal medicines as assessed in vitro: a review. Chem Biodivers 20:e202201098 36595710 10.1002/cbdv.202201098

[CR55] Ge D, Luo T, Sun Y, Liu M, Lyu Y, Yin W, Li R, Zhang Y, Yue H, Liu N (2024) Natural diterpenoid EKO activates deubiqutinase ATXN3 to preserve vascular endothelial integrity and alleviate diabetic retinopathy through c-fos/focal adhesion axis. Int J Biol Macromol 260:129341 38218272 10.1016/j.ijbiomac.2024.129341

[CR56] Georgiou M, Robson AG, Fujinami K, de Guimarães TAC, Fujinami-Yokokawa Y, Varela MD, Pontikos N, Kalitzeos A, Mahroo OA, Webster AR (2024) Phenotyping and genotyping inherited retinal diseases: molecular genetics, clinical and imaging features, and therapeutics of macular dystrophies, cone and cone-rod dystrophies, rod-cone dystrophies, leber congenital amaurosis, and cone dysfunction syndromes. Prog Retin Eye Res 100:101244. 10.1016/j.preteyeres.2024.101244 10.1016/j.preteyeres.2024.101244 38278208

[CR57] González P, Lozano P, Ros G, Solano F, González P, Lozano P, Ros G, Solano F (2023) Hyperglycemia and oxidative stress: an integral, updated and critical overview of their metabolic interconnections. Int J Mol Sci 24:9352 37298303 10.3390/ijms24119352 PMC10253853

[CR58] Gui S, Tang W, Huang Z, Wang X, Gui S, Gao X, Xiao D, Tao L, Jiang Z, Wang X (2023) Ultrasmall coordination polymer nanodots Fe-Quer nanozymes for preventing and delaying the development and progression of diabetic retinopathy. Adv Func Mater 33:2300261

[CR59] Gupta SK, Kumar B, Nag TC, Srinivasan B, Srivastava S, Gaur S, Saxena R (2014) Effects of Trigonella foenum-graecum (L.) on retinal oxidative stress, and proinflammatory and angiogenic molecular biomarkers in streptozotocin-induced diabetic rats. Mol Cell Biochem 388:1–9 24242137 10.1007/s11010-013-1893-2

[CR60] Han J-X, Wang H, Liang H-H, Guo J-X (2021) Correlation of the retinopathy degree with the change of ocular surface and corneal nerve in patients with type 2 diabetes mellitus. Int J Ophthalmol 14:750 34012892 10.18240/ijo.2021.05.17 PMC8077005

[CR61] Hsieh M-C, Cheng C-Y, Li K-H, Chuang C-C, Wu J-S, Lee S-T, Lu W-Y, Chiu S-L, Liu Y-L, Chen S-N (2022) Diabetic macular edema and proliferative diabetic retinopathy treated with anti-vascular endothelial growth factor under the reimbursement policy in Taiwan. Sci Rep 12:711 35027613 10.1038/s41598-021-04593-x PMC8758685

[CR62] Huang J, Yi Q, You Y, Chen Y, Niu T, Li Y, Zhang J, Ji X, Xu G, Zou W (2021a) Curcumin suppresses oxidative stress via regulation of ROS/NF-κB signaling pathway to protect retinal vascular endothelial cell in diabetic retinopathy. Mol Cell Toxicol 17:367–376

[CR63] Huang S-Y, Jeng C, Kao S-C, Yu JJ-H, Liu D-Z (2004) Improved haemorrheological properties by Ginkgo biloba extract (Egb 761) in type 2 diabetes mellitus complicated with retinopathy. Clin Nutr 23:615–621 15297098 10.1016/j.clnu.2003.10.010

[CR64] Huang Y, Wang Z, Ma H, Ji S, Chen Z, Cui Z, Chen J, Tang S (2021b) Dysbiosis and implication of the gut microbiota in diabetic retinopathy. Front Cell Infect Microbiol 11:646348 33816351 10.3389/fcimb.2021.646348 PMC8017229

[CR65] Iglicki M, González DP, Loewenstein A, Zur D (2022) Next-generation anti-VEGF agents for diabetic macular oedema. Eye 36:273–277 34373607 10.1038/s41433-021-01722-8 PMC8807622

[CR66] Jin Q, Hao X-F, Xie L-K, Xu J, Sun M, Yuan H, Wang S-H, Wu G-P, Miao M-L (2020) A network pharmacology to explore the mechanism of Astragalus Membranaceus in the treatment of diabetic retinopathy. Evid-Based Complement Alternat Med 2020:8878569 33204295 10.1155/2020/8878569 PMC7652614

[CR67] Kamalu AO, Ekeoba AE, Uzor EC, Duru CC, Anyatonwu OP, Adiele OE, Amuzie CR, Odoemenam CL (2024) Mitigating the prevalence of diabetic retinopathy in the United States: utilization of the chronic care model as a public health framework. Open J Ophthalmol 14:103–116

[CR68] Kang Q, Yang C (2020) Oxidative stress and diabetic retinopathy: molecular mechanisms, pathogenetic role and therapeutic implications. Redox Biol 37:101799 33248932 10.1016/j.redox.2020.101799 PMC7767789

[CR69] Kharbach M, Marmouzi I, El Jemli M, Bouklouze A, Vander Heyden Y (2020) Recent advances in untargeted and targeted approaches applied in herbal-extracts and essential-oils fingerprinting-a review. J Pharm Biomed Anal 177:112849 31499429 10.1016/j.jpba.2019.112849

[CR70] Kim J, Kim C-S, Lee YM, Sohn E, Jo K, Kim JS (2015) Vaccinium myrtillus extract prevents or delays the onset of diabetes–induced blood–retinal barrier breakdown. Int J Food Sci Nutr 66:236–242 25582181 10.3109/09637486.2014.979319

[CR71] Kocaadam B, Şanlier N (2017) Curcumin, an active component of turmeric (Curcuma longa), and its effects on health. Crit Rev Food Sci Nutr 57:2889–2895 26528921 10.1080/10408398.2015.1077195

[CR72] Kowluru RA, Menon B, Gierhart DL (2008) Beneficial effect of zeaxanthin on retinal metabolic abnormalities in diabetic rats. Invest Ophthalmol vis Sci 49:1645–1651 18385086 10.1167/iovs.07-0764

[CR73] Kropp M, Golubnitschaja O, Mazurakova A, Koklesova L, Sargheini N, Vo T-TKS, de Clerck E, Polivka J Jr, Potuznik P, Polivka J (2023) Diabetic retinopathy as the leading cause of blindness and early predictor of cascading complications—risks and mitigation. Epma J 14:21–42 36866156 10.1007/s13167-023-00314-8 PMC9971534

[CR74] Kumar S, Mittal A, Babu D, Mittal A (2021) Herbal medicines for diabetes management and its secondary complications. Curr Diabetes Rev 17:437–456 33143632 10.2174/1573399816666201103143225

[CR75] Kwok SS, Bu Y, Lo AC-Y, Chan TC-Y, So KF, Lai JS-M, Shih KC (2019) A systematic review of potential therapeutic use of Lycium barbarum polysaccharides in disease. Biomed Res Int 2019:4615745 30891458 10.1155/2019/4615745 PMC6390233

[CR76] Lachin JM, Nathan DM (2021) Understanding metabolic memory: the prolonged influence of glycemia during the diabetes control and complications trial (DCCT) on future risks of complications during the study of the epidemiology of diabetes interventions and complications (EDIC). Diabetes Care 44:2216–2224 34548284 10.2337/dc20-3097 PMC8929187

[CR77] Laddha UD, Kshirsagar SJ (2020) Formulation of PPAR-gamma agonist as surface modified PLGA nanoparticles for non-invasive treatment of diabetic retinopathy: in vitro and in vivo evidences. Heliyon 6:e04589 32832706 10.1016/j.heliyon.2020.e04589 PMC7432955

[CR78] Lahoti S, Nashawi M, Sheikh O, Massop D, Mir M, Chilton R (2021) Sodium-glucose co-transporter 2 inhibitors and diabetic retinopathy: insights into preservation of sight and looking beyond. Cardiovasc Endocrinol Metab 10:3–13 33634250 10.1097/XCE.0000000000000209 PMC7901818

[CR79] Lai FHP, Wong EWN, Lam WC, Lee TC, Wong SC, Nagiel A, Lam RF (2021) Endoscopic vitreoretinal surgery: review of current applications and future trends. Surv Ophthalmol 66:198–212 33278403 10.1016/j.survophthal.2020.11.004

[CR80] Lange R, Kumagai A, Weiss S, Zaffke KB, Day S, Wicker D, Howson A, Jayasundera KT, Smolinski L, Hedlich C (2021) Vision-related quality of life in adults with severe peripheral vision loss: a qualitative interview study. J Patient-Rep Outcomes 5:1–12 33439361 10.1186/s41687-020-00281-y PMC7806695

[CR81] Lanzani G, Chiaravalli G, Colombo E, Manfredi G, Di Marco S, Vurro V, Benfenati F (2024) Nanotechnology for vision restoration. Nat Rev Bioeng 2:829–848

[CR82] Li J, Liu Y, Geng K, Lu X, Shen X, Guo Q (2024a) ROS-responsive nanoparticles with antioxidative effect for the treatment of diabetic retinopathy. J Biomater Sci Polym Ed 1–22. 10.1080/09205063.2024.2406628 10.1080/09205063.2024.2406628 39316729

[CR83] Li J, Lv P, Xiao Z, Xiao J (2024b) Protective effects of bioactive compound-derived nanoparticle against diabetic retinopathy through the modulation of the NF-κB signaling pathway. ACS Omega 9:26267–26274 38911745 10.1021/acsomega.4c02066 PMC11191572

[CR84] Li J, Zhao T, Sun Y (2024c) Interleukin-17A in diabetic retinopathy: the crosstalk of inflammation and angiogenesis. Biochem Pharmacol 225:116311 38788958 10.1016/j.bcp.2024.116311

[CR85] Li R, Du S, Ye Z, Yang W, Ding Z, Liu Y (2024d) Protective effect of cyanidin-3-O-glucoside from blueberry anthocyanin extracts against hyperglycemia-induced outer BRB damage by suppressing REDD1-mediated VEGFA upregulation. Food Biosci 61:104695

[CR86] Li Y, Wang K, Zhu X, Cheng Z, Zhu L, Murray M, Zhou F (2023) Ginkgo biloba extracts protect human retinal Müller glial cells from t-BHP induced oxidative damage by activating the AMPK-Nrf2-NQO-1 axis. J Pharm Pharmacol 75:385–396 36583518 10.1093/jpp/rgac095

[CR87] Liao W-L, Huang C-P, Wang H-P, Lei Y-J, Lin H-J, Huang Y-C (2023) Diosgenin, a natural steroidal sapogenin, alleviates the progression of diabetic retinopathy in diabetic mice. In Vivo 37: 661–666 10.21873/invivo.13126 PMC10026653 36881067

[CR88] Lindner M, Arefnia B, Ivastinovic D, Sourij H, Lindner E, Wimmer G (2022) Association of periodontitis and diabetic macular edema in various stages of diabetic retinopathy. Clin Oral Invest 26:505–512 10.1007/s00784-021-04028-x PMC8791870 34159405

[CR89] Liu J, Guo B, Liu Q, Zhu G, Wang Y, Wang N, Yang Y, Fu S, Liu J, Guo B, Liu Q, Zhu G, Wang Y, Wang N, Yang Y, Fu S (2024) Cellular senescence: a bridge between diabetes and microangiopathy. Biomolecules 14:1361 39595537 10.3390/biom14111361 PMC11591988

[CR90] Liu Z, Yu X (2024) Development of a T2D app for elderly users: participatory design study via heuristic evaluation and usability testing. Electronics 13:3862

[CR91] López-Contreras AK, Martínez-Ruiz MG, Olvera-Montaño C, Robles-Rivera RR, Arévalo-Simental DE, Castellanos-González JA, Hernández-Chávez A, Huerta-Olvera SG, Cardona-Muñoz EG, Rodríguez-Carrizalez AD (2020) Importance of the use of oxidative stress biomarkers and inflammatory profile in aqueous and vitreous humor in diabetic retinopathy. Antioxidants 9:891 32962301 10.3390/antiox9090891 PMC7555116

[CR92] Lotfi M, Butler AE, Sukhorukov VN, Sahebkar A (2024) Application of CRISPR-Cas9 technology in diabetes research. Diabet Med 41:e15240 37833064 10.1111/dme.15240

[CR93] Lu AQ, Rizk M, O’rourke T, Goodling K, Lehman E, Scott IU, Pantanelli SM (2022) Safety and efficacy of topical vs intracanalicular corticosteroids for the prevention of postoperative inflammation after cataract surgery. J Cataract Refract Surg 48:1242–1247 35537939 10.1097/j.jcrs.0000000000000963 PMC9622372

[CR94] Madit W, Harnirattisai T, Hain D, Gaudio PA (2024) Effect of a self-care promoting program on engagement in self-care behaviors and health-related outcomes among persons with type 2 diabetes and diabetic retinopathy: a single-blind randomized controlled trial. Belitung Nurs J 10:272 38947309 10.33546/bnj.3360 PMC11211747

[CR95] Maeng Y-S, Maharjan S, Kim J-H, Park J-H, Suk YuY, Kim Y-M, Kwon Y-G (2013) Rk1, a ginsenoside, is a new blocker of vascular leakage acting through actin structure remodeling. PLoS ONE 8:e68659 23894330 10.1371/journal.pone.0068659 PMC3718811

[CR96] Martínez-Solís I, Acero N, Bosch-Morell F, Castillo E, González-Rosende ME, Muñoz-Mingarro D, Ortega T, Sanahuja MA, Villagrasa V (2019) Neuroprotective potential of Ginkgo biloba in retinal diseases. Planta Med 85:1292–1303 31266069 10.1055/a-0947-5712

[CR97] Matos AL, Bruno DF, Ambrósio AF, Santos PF (2020) The benefits of flavonoids in diabetic retinopathy. Nutrients 12:3169 33081260 10.3390/nu12103169 PMC7603001

[CR98] Matsuno H, Tsuchimine S, O’Hashi K, Sakai K, Hattori K, Hidese S, Nakajima S, Chiba S, Yoshimura A, Fukuzato N (2022) Association between vascular endothelial growth factor-mediated blood–brain barrier dysfunction and stress-induced depression. Mol Psychiatry 27:3822–3832 35618888 10.1038/s41380-022-01618-3

[CR99] Mishra MK (2024) A comprehensive evaluation of the herbal role in the management of diabetic retinopathy. AfrJBioSc 6. 10.48047/AFJBS.6.12.2024.5960-5975

[CR100] Moldovan M, Păpurică A-M, Muntean M, Bungărdean RM, Gheban D, Moldovan B, Katona G, David L, Filip GA (2023) Effects of gold nanoparticles phytoreduced with rutin in an early rat model of diabetic retinopathy and cataracts. Metabolites 13:955 37623898 10.3390/metabo13080955 PMC10456405

[CR101] Mossburg S, Kilany M, Jinnett K, Nguyen C, Soles E, Wood-Palmer D, Aly M (2024) A rapid review of interventions to improve care for people who are medically underserved with multiple sclerosis, diabetic retinopathy, and lung cancer. Int J Environ Res Public Health 21:529 38791744 10.3390/ijerph21050529 PMC11121396

[CR102] Mrowicka M, Mrowicki J, Majsterek I (2024) Relationship between biochemical pathways and non-coding RNAs involved in the progression of diabetic retinopathy. J Clin Med 13:292 38202299 10.3390/jcm13010292 PMC10779474

[CR103] Mrugacz M, Bryl A, Zorena K (2021) Retinal vascular endothelial cell dysfunction and neuroretinal degeneration in diabetic patients. J Clin Med 10:458 33504108 10.3390/jcm10030458 PMC7866162

[CR104] Mykkänen OT, Kalesnykas G, Adriaens M, Evelo CT, Törrönen R, Kaarniranta K (2012) Bilberries potentially alleviate stress-related retinal gene expression induced by a high-fat diet in mice. Mol vis 18:2338 22993483 PMC3444297

[CR105] Nakajima J-i, Tanaka I, Seo S, Yamazaki M, Saito K (2004) LC/PDA/ESI-MS profiling and radical scavenging activity of anthocyanins in various berries. Biomed Res Int 2004:241–247 10.1155/S1110724304404045 PMC1082896 15577184

[CR106] Ng SM, Ayoola OO, McGuigan M, Chandrasekaran S (2019) A multicentre study evaluating the risk and prevalence of diabetic retinopathy in children and young people with type 1 diabetes mellitus. Diabetes Metab Syndr 13:744–746 30641800 10.1016/j.dsx.2018.11.063

[CR107] Nian S, Lo ACY, Mi Y, Ren K, Yang D, Nian S, Lo ACY, Mi Y, Ren K, Yang D (2021) Neurovascular unit in diabetic retinopathy: pathophysiological roles and potential therapeutical targets. Eye Vision 8:15 33931128 10.1186/s40662-021-00239-1 PMC8088070

[CR108] Nichani PA, Popovic MM, Dhoot AS, Pathak A, Muni RH, Kertes PJ (2023) Treat-and-extend dosing of intravitreal anti-VEGF agents in neovascular age-related macular degeneration: a meta-analysis. Eye 37:2855–2863 36859600 10.1038/s41433-023-02439-6 PMC10517126

[CR109] O’Leary F, Campbell M (2023) The blood–retina barrier in health and disease. FEBS J 290:878–891 34923749 10.1111/febs.16330

[CR110] Oboh RA, Precious LB (2024) Visual and Psychological challenges associated with geriatric ocular disorders among patients visiting the University of Port Harcourt Teaching Hospital, Rivers State Nigeria. Med Sci Res Bull 1:14–25

[CR111] Orji A, Rani PK, Narayanan R, Sahoo NK, Das T (2021) The economic burden of diabetic retinopathy care at a tertiary eye care center in South India. Indian J Ophthalmol 69:666–670 33595498 10.4103/ijo.IJO_1538_20 PMC7942062

[CR112] Oshitari T (2024) Translational research and therapies for neuroprotection and regeneration of the optic nerve and retina: a narrative review. Int J Mol Sci 25:10485 39408817 10.3390/ijms251910485 PMC11476551

[CR113] Pandya M, Banait S, Daigavane S (2024) Insights into visual rehabilitation: pan-retinal photocoagulation for proliferative diabetic retinopathy. Cureus 16:e54273 38496130 10.7759/cureus.54273 PMC10944551

[CR114] Parashar R, Vyas A, Sah AK, Hemnani N, Thangaraju P, Suresh PK (2024) Recent updates on nanocarriers for drug delivery in posterior segment diseases with emphasis on diabetic retinopathy. Curr Diabetes Rev 20:1–24 37855359 10.2174/0115733998240053231009060654

[CR115] Park SY, Park K, Oh J-W, Park G (2023) Gold nanoparticle encoded with marigold (Tagetes erecta L.) suppressed hyperglycemia-induced senescence in retinal pigment epithelium via suppression of lipid peroxidation. Arab J Chem 16:105120

[CR116] Parravano M, Costanzo E, Sacconi R, Querques G (2023) DRCR. net trials for diabetic macular edema. Diabetic Macular Edema. Springer, Singapore, pp 69–75. 10.1007/978-981-19-7307-9_8

[CR117] Patel NC, Hsieh Y-T, Yang C-M, Berrocal MH, Dhawahir-Scala F, Ruamviboonsuk P, Pappuru RR, Dave VP (2024) Vitrectomy for diabetic retinopathy: a review of indications, techniques, outcomes, and complications. Taiwan J Ophthalmol 14:519–530 39803397 10.4103/tjo.TJO-D-23-00108 PMC11717329

[CR118] Pawar SD, Gawali K, Jat S, Singh P, Datusalia AK, Kulhari H, Kumar P (2024) Physiochemical characterization and pharmacokinetic assessment of Bergamottin solid lipid nanoparticles. J Drug Deliv Sci Technol 93:105426

[CR119] Pei X, Huang D, Li Z (2024) Genetic insights and emerging therapeutics in diabetic retinopathy: from molecular pathways to personalized medicine. Front Genet 15:1416924 39246572 10.3389/fgene.2024.1416924 PMC11378321

[CR120] Pellegrini M, Senni C, Bernabei F, Cicero AFG, Vagge A, Maestri A, Scorcia V, Giannaccare G (2020) The role of nutrition and nutritional supplements in ocular surface diseases. Nutrients 12:952 32235501 10.3390/nu12040952 PMC7230622

[CR121] Pirmardvand Chegini S, Varshosaz J, Dehghani A, Minaiyan M, Mirmohammad Sadeghi H (2022) Ocular delivery of sunitinib-loaded nanoparticles doped in tragacanthic acid hydrogel in treatment of diabetic retinopathy in rats. Drug Dev Ind Pharm 48:29–39 35723593 10.1080/03639045.2022.2092745

[CR122] Qu Q, Park K, Zhou K, Wassel D, Farjo R, Criswell T, Ma J-x, Zhang Y (2022) Sustained therapeutic effect of an anti-inflammatory peptide encapsulated in nanoparticles on ocular vascular leakage in diabetic retinopathy. Front Cell Dev Biol 10:1049678 36589744 10.3389/fcell.2022.1049678 PMC9802579

[CR123] Radwan SE-S, El-Kamel A, Zaki EI, Burgalassi S, Zucchetti E, El-Moslemany RM (2021) Hyaluronic-coated albumin nanoparticles for the non-invasive delivery of apatinib in diabetic retinopathy. Int J Nanomed 16:4481–4494 10.2147/IJN.S316564 PMC8259843 34239300

[CR124] Rajpal A, Rahimi L, Ismail-Beigi F (2020) Factors leading to high morbidity and mortality of COVID-19 in patients with type 2 diabetes. J Diabetes 12:895–908 32671936 10.1111/1753-0407.13085 PMC7405270

[CR125] Reddy SK, Devi V, Seetharaman ATM, Shailaja S, Bhat KMR, Gangaraju R, Upadhya D (2024) Cell and molecular targeted therapies for diabetic retinopathy. Front Endocrinol 15:1416668 10.3389/fendo.2024.1416668 PMC11211264 38948520

[CR126] Ristori E (2021) Vascular endothelial growth factors and endothelial cells behaviour. Department of Biotechnology, Chemistry and Pharmacy. UNIVERSITÀ DI SIENA. 10.25434/ristori-emma_phd2021

[CR127] Rizwan S, Kumar KVP (2024) A highly compact intra-ocular antenna characterization for retinal prosthesis application. 2024 International Conference on Recent Advances in Electrical, Electronics, Ubiquitous Communication, and Computational Intelligence (RAEEUCCI). IEEE, pp. 1–5. 10.1109/RAEEUCCI61380.2024.10547988

[CR128] Rodríguez ML, Pérez S, Mena-Mollá S, Desco MC, Ortega ÁL (2019) Oxidative stress and microvascular alterations in diabetic retinopathy: future therapies. Oxid Med Cell Longev 2019:4940825 31814880 10.1155/2019/4940825 PMC6878793

[CR129] Rohilla M, Bansal S, Garg A, Dhiman S, Dhankhar S, Saini M, Chauhan S, Alsubaie N, Batiha GE-S, Albezrah NKA (2023) Discussing pathologic mechanisms of diabetic retinopathy & therapeutic potentials of curcumin and β-glucogallin in the management of Diabetic retinopathy. Biomed Pharmacother 169:115881 37989030 10.1016/j.biopha.2023.115881

[CR130] Rojas A, Lindner C, Schneider I, Gonzalez I, Uribarri J, Rojas A, Lindner C, Schneider I, Gonzalez I, Uribarri J (2024) The RAGE axis: a relevant inflammatory hub in human diseases. Biomolecules 14:412 38672429 10.3390/biom14040412 PMC11048448

[CR131] Rowe LW, Ciulla TA (2024) Gene therapy for non-hereditary retinal disease: age-related macular degeneration, diabetic retinopathy, and beyond. Genes 15:720 38927656 10.3390/genes15060720 PMC11203163

[CR132] Rossino MG, Casini G (2019) Nutraceuticals for the treatment of diabetic retinopathy. Nutrients 11:771 30987058 10.3390/nu11040771 PMC6520779

[CR133] Rudraraju M, Narayanan SP, Somanath PR (2020) Regulation of blood-retinal barrier cell-junctions in diabetic retinopathy. Pharmacol Res 161:105115 32750417 10.1016/j.phrs.2020.105115 PMC7755666

[CR134] Sachan A, Singh A, Shukla S, Aggarwal S, Mir I, Yadav R (2020) Serum adipocytokines levels and their association with insulin sensitivity in morbidly obese individuals undergoing bariatric surgery. J Obes Metab Syndr 29:303 33380577 10.7570/jomes20090 PMC7789018

[CR135] Sam S (2019) Importance and effectiveness of herbal medicines. J Pharmacogn Phytochem 8:354–357

[CR136] Sanders JP, Daley AJ, Esliger DW, Roalfe AK, Colda A, Turner J, Hajdu S, Potter A, Humayun AM, Spiliotis I (2024) Effectiveness of a digital health and financial incentive intervention to promote physical activity in patients with type 2 diabetes: study protocol for a randomised controlled trial with a nested qualitative study—ACTIVATE trial. Trials 25:755 39533314 10.1186/s13063-024-08513-y PMC11559103

[CR137] Sasaki M, Yuki K, Hanyuda A, Yamagishi K, Motomura K, Kurihara T, Tomita Y, Mori K, Ozawa N, Ozawa Y (2023) Associations between fatty acid intake and diabetic retinopathy in a Japanese population. Sci Rep 13:12903 37558714 10.1038/s41598-023-39734-x PMC10412616

[CR138] Schreur V, van Asten F, Ng H, Weeda J, Groenewoud JM, Tack CJ, Hoyng CB, de Jong EK, Klaver CC, Jeroen Klevering B (2018) Risk factors for development and progression of diabetic retinopathy in Dutch patients with type 1 diabetes mellitus. Acta Ophthalmol 96:459–464 30188024 10.1111/aos.13815 PMC6174939

[CR139] Sen S, Chen S, Wu Y, Feng B, Lui EK, Chakrabarti S (2013) Preventive effects of North American ginseng (Panax quinquefolius) on diabetic retinopathy and cardiomyopathy. Phytother Res 27:290–298 22566158 10.1002/ptr.4719

[CR140] Senthil S, Dada T, Das T, Kaushik S, Puthuran GV, Philip R, Rani PK, Rao H, Singla S, Vijaya L (2021) Neovascular glaucoma-a review. Indian J Ophthalmol 69:525–534 33595466 10.4103/ijo.IJO_1591_20 PMC7942095

[CR141] Sepahi S, Soheili Z-S, Tavakkol-Afshari J, Mehri S, Hosseini SM, Mohajeri SA, Khodaverdi E (2021) Retinoprotective effects of crocin and crocetin via anti-angiogenic mechanism in high glucose-induced human retinal pigment epithelium cells. Curr Mol Pharmacol 14:883–893 33881975 10.2174/1874467214666210420111232

[CR142] Shah J, Cheong ZY, Tan B, Wong D, Liu X, Chua J (2022) Dietary intake and diabetic retinopathy: a systematic review of the literature. Nutrients 14:5021 36501054 10.3390/nu14235021 PMC9735534

[CR143] Shaikh N, Srishti R, Khanum A, Thirumalesh MB, Dave V, Arora A, Bansal R, Surve A, Azad S, Kumar V (2023) Vitreous hemorrhage–causes, diagnosis, and management. Indian J Ophthalmol 71:28–38 36588205 10.4103/ijo.IJO_928_22 PMC10155538

[CR144] Shazly TA, Latina MA (2009) Neovascular glaucoma: etiology, diagnosis and prognosis. Semin Ophthal 24(2):113–21. 10.1080/08820530902800801 10.1080/08820530902800801 19373696

[CR145] Shekar BRC, Nagarajappa R, Suma S, Thakur R (2015) Herbal extracts in oral health care-a review of the current scenario and its future needs. Pharmacogn Rev 9:87 26392704 10.4103/0973-7847.162101 PMC4557240

[CR146] Silpa-archa S, Tadarati M, Chotcomwongse P, Ruamviboonsuk P (2024) Diabetic retinopathy and choroidopathy: pathophysiology, imaging findings, and treatment updates. Retinal and Choroidal Vascular Diseases of the Eye. Elsevier, pp 227–247

[CR147] Sivaprasad S, Gupta B, Gulliford MC, Dodhia H, Mohamed M, Nagi D, Evans JR (2012) Ethnic variations in the prevalence of diabetic retinopathy in people with diabetes attending screening in the United Kingdom (DRIVE UK). PLoS ONE 7:e32182 22412857 10.1371/journal.pone.0032182 PMC3297598

[CR148] Skourtis G, Krontira A, Ntaoula S, Ferlemi AV, Zeliou K, Georgakopoulos C, Margarity GM, Lamari NF, Pharmakakis N (2020) Protective antioxidant effects of saffron extract on retinas of streptozotocin-induced diabetic rats. Rom J Ophthalmol 64:394 33367177 10.22336/rjo.2020.61 PMC7739020

[CR149] Soares NP, Magalhaes GC, Mayrink PH, Verano-Braga T (2024) Omics to unveil diabetes mellitus pathogenesis and biomarkers: focus on proteomics, lipidomics, and metabolomics. Mass Spectrometry-Based Approaches for Treating Human Diseases and Diagnostics, pp 211–220. 10.1007/978-3-031-50624-6_11 10.1007/978-3-031-50624-6_11 38409423

[CR150] Song MK, Roufogalis BD, Huang TH (2012) Modulation of diabetic retinopathy pathophysiology by natural medicines through PPAR-γ-related pharmacology. Br J Pharmacol 165:4–19 21480863 10.1111/j.1476-5381.2011.01411.x PMC3252962

[CR151] Song P, Yu J, Chan KY, Theodoratou E, Rudan I (2018) Prevalence, risk factors and burden of diabetic retinopathy in China: a systematic review and meta-analysis. J Glob Health 8:010803 29899983 10.7189/jogh.08.010803 PMC5997368

[CR152] Song Y, Huang L, Yu J (2016) Effects of blueberry anthocyanins on retinal oxidative stress and inflammation in diabetes through Nrf2/HO-1 signaling. J Neuroimmunol 301:1–6 27847126 10.1016/j.jneuroim.2016.11.001

[CR153] Spooner KL, Fraser-Bell S, Hong T, Wong JG, Chang AA (2021) Long-term outcomes of anti-VEGF treatment of retinal vein occlusion. Eye 36:1194–1201 34117379 10.1038/s41433-021-01620-z PMC9151794

[CR154] Srejovic JV, Muric MD, Jakovljevic VL, Srejovic IM, Sreckovic SB, Petrovic NT, Todorovic DZ, Bolevich SB, Sarenac Vulovic TS (2024) Molecular and cellular mechanisms involved in the pathophysiology of retinal vascular disease—interplay between inflammation and oxidative stress. Int J Mol Sci 25:1185. 10.3390/ijms252111850 39519401 10.3390/ijms252111850 PMC11546760

[CR155] Stoia D, Nistor M, Suciu M, Borlan R, Campu A, Rugina D, Maniu D, Astilean S, Focsan M (2023) NIR photothermal-activable drug-conjugated microcapsules for in vitro targeted delivery and release: an alternative treatment of diabetic retinopathy. Int J Pharm 635:122700 36764419 10.1016/j.ijpharm.2023.122700

[CR156] Su L, Hong Z, Zhou T, Jian Y, Xu M, Zhang X, Zhu X, Wang J (2022) Health improvements of type 2 diabetic patients through diet and diet plus fecal microbiota transplantation. Sci Rep 12:1152 35064189 10.1038/s41598-022-05127-9 PMC8782834

[CR157] Sun H-Q, Zhou Z-Y (2010) Effect of ginsenoside-Rg3 on the expression of VEGF and TNF-α in retina with diabetic rats. Int J Ophthalmol 3:220 22553558 10.3980/j.issn.2222-3959.2010.03.09 PMC3340625

[CR158] Sun K, Chen Y, Zheng S, Wan W, Hu K (2024) Genipin ameliorates diabetic retinopathy via the HIF-1α and AGEs-RAGE pathways. Phytomedicine 129:155596 38626646 10.1016/j.phymed.2024.155596

[CR159] Sun W-J, An X-D, Zhang Y-H, Zhao X-F, Sun Y-T, Yang C-Q, Kang X-M, Jiang L-L, Ji H-Y, Lian F-M (2023) The ideal treatment timing for diabetic retinopathy: the molecular pathological mechanisms underlying early-stage diabetic retinopathy are a matter of concern. Front Endocrinol 14:1270145 10.3389/fendo.2023.1270145 PMC10680169 38027131

[CR160] Taheri SL, Rezazadeh M, Hassanzadeh F, Akbari V, Dehghani A, Talebi A, Mostafavi SA (2022) Preparation, physicochemical, and retinal anti-angiogenic evaluation of poloxamer hydrogel containing dexamethasone/avastin-loaded chitosan-N-acetyl-L-cysteine nanoparticles. Int J Biol Macromol 220:1605–1618 36116595 10.1016/j.ijbiomac.2022.09.101

[CR161] Takayama K, Someya H, Yokoyama H, Takamura Y, Morioka M, Sameshima S, Ueda T, Kitano S, Tashiro M, Sugimoto M (2019) Risk factors of neovascular glaucoma after 25-gauge vitrectomy for proliferative diabetic retinopathy with vitreous hemorrhage: a retrospective multicenter study. Sci Rep 9:14858 31619708 10.1038/s41598-019-51411-6 PMC6795876

[CR162] Taloni A, Coco G, Rastelli D, Buffon G, Scorcia V, Giannaccare G (2023) Safety and efficacy of dexamethasone intravitreal implant given either first-line or second-line in diabetic macular edema. Patient Preference Adherence 17:3307–3329. 10.2147/PPA.S427209 38106365 10.2147/PPA.S427209 PMC10725633

[CR163] Tan GS-W, Chakravarthy U, Wong TY (2020) Anti-VEGF therapy or vitrectomy surgery for vitreous hemorrhage from proliferative diabetic retinopathy. JAMA 324:2375–2377 33320207 10.1001/jama.2020.22829

[CR164] Tang L, Xu G-T, Zhang J-F (2023) Inflammation in diabetic retinopathy: possible roles in pathogenesis and potential implications for therapy. Neural Regen Res 18:976–982 36254977 10.4103/1673-5374.355743 PMC9827774

[CR165] Tang L, Zhang Y, Jiang Y, Willard L, Ortiz E, Wark L, Medeiros D, Lin D (2011) Dietary wolfberry ameliorates retinal structure abnormalities in db/db mice at the early stage of diabetes. Exp Biol Med 236:1051–1063 10.1258/ebm.2011.010400 PMC3166358 21750018

[CR166] Tang S, An X, Sun W, Zhang Y, Yang C, Kang X, Sun Y, Jiang L, Zhao X, Gao Q (2024) Parallelism and non-parallelism in diabetic nephropathy and diabetic retinopathy. Front Endocrinol 15:1336123 10.3389/fendo.2024.1336123 PMC10899692 38419958

[CR167] Teo ZL, Tham Y-C, Yu M, Chee ML, Rim TH, Cheung N, Bikbov MM, Wang YX, Tang Y, Lu Y (2021) Global prevalence of diabetic retinopathy and projection of burden through 2045: systematic review and meta-analysis. Ophthalmology 128:1580–1591 33940045 10.1016/j.ophtha.2021.04.027

[CR168] Thangarajah H, Vial IN, Grogan RH, Yao D, Shi Y, Januszyk M, Galiano RD, Chang EI, Galvez MG, Glotzbach JP, Wong VW, Brownlee M, Gurtner GC (2010) HIF-1α dysfunction in diabetes. Cell Cycle 9(1):75–9. 10.4161/cc.9.1.10371 10.4161/cc.9.1.10371 20016290

[CR169] Tsung T-H, Chen Y-H, Lu D-W (2023) Updates on biodegradable formulations for ocular drug delivery. Pharmaceutics 15:734 36986595 10.3390/pharmaceutics15030734 PMC10057390

[CR170] Uludag G, Hassan M, Matsumiya W, Pham BH, Chea S, Trong Tuong Than N, Doan HL, Akhavanrezayat A, Halim MS, Do DV (2022) Efficacy and safety of intravitreal anti-VEGF therapy in diabetic retinopathy: what we have learned and what should we learn further? Expert Opin Biol Ther 22:1275–1291 35818801 10.1080/14712598.2022.2100694 PMC10863998

[CR171] ValdezGuerrero AS, Quintana-Pérez JC, Arellano-Mendoza MG, Castañeda-Ibarra FJ, Tamay-Cach F, Alemán-González-Duhart D (2021) Diabetic retinopathy: important biochemical alterations and the main treatment strategies. Can J Diabetes 45:504–511 33341391 10.1016/j.jcjd.2020.10.009

[CR172] Vaneková Z, Rollinger JM (2022) Bilberries: Curative and miraculous–a review on bioactive constituents and clinical research. Front Pharmacol 13:909914 35847049 10.3389/fphar.2022.909914 PMC9277355

[CR173] Varghese DS, Ali BR (2021) Pathological crosstalk between oxidized LDL and ER stress in human diseases: a comprehensive review. Front Cell Dev Biol 9:674103 34124059 10.3389/fcell.2021.674103 PMC8187772

[CR174] Wal P, Wal A, Gupta D, Mantry S, Mahajan KC, Rathore S, Behl T (2024) Diabetic retinopathy: stressing the function of angiogenesis, inflammation and oxidative stress. Targeting Angiogenesis, Inflammation, and Oxidative Stress in Chronic Diseases, Academic Press, pp 323–348. 10.1016/B978-0-443-13587-3.00002-3

[CR175] Wang C, Wang K, Li P (2022) Blueberry anthocyanins extract attenuated diabetic retinopathy by inhibiting endoplasmic reticulum stress via the miR-182/OGG1 axis. J Pharmacol Sci 150:31–40 35926946 10.1016/j.jphs.2022.06.004

[CR176] Wang D-d, Zhu H-z, Li S-w, Yang J-m, Xiao Y, Kang Q-r, Li C-y, Zhao Y-s, Zeng Y, Li Y (2016) Crude saponins of Panax notoginseng have neuroprotective effects to inhibit palmitate-triggered endoplasmic reticulum stress-associated apoptosis and loss of postsynaptic proteins in staurosporine differentiated RGC-5 retinal ganglion cells. J Agric Food Chem 64:1528–1539 26832452 10.1021/acs.jafc.5b05864

[CR177] Wang S, Du S, Wang W, Zhang F (2020) Therapeutic investigation of quercetin nanomedicine in a zebrafish model of diabetic retinopathy. Biomed Pharmacother 130:110573 32745912 10.1016/j.biopha.2020.110573

[CR178] Wang S, Hua R, Zhao Y, Liu L (2024a) Laser treatment for diabetic retinopathy: history, mechanism, and novel technologies. J Clin Med 13:5439 39336925 10.3390/jcm13185439 PMC11432231

[CR179] Wang Y, Gao S, Cao F, Yang H, Lei F, Hou S (2024b) Ocular immune-related diseases: molecular mechanisms and therapy. MedComm 5:e70021. 10.1002/mco2.70021 39611043 10.1002/mco2.70021 PMC11604294

[CR180] Wilkins CS, Sobol EK, Lema GM, Lee JG, Rosen RB, Deobhakta A (2022) Intravitreal dexamethasone insert in diabetic macular edema super-refractory to anti-vascular endothelial growth factor therapy. Eur J Ophthalmol 32:NP37-NP41 10.1177/11206721211004391 33757333

[CR181] Wiszniak S, Schwarz Q, Wiszniak S, Schwarz Q (2021) Exploring the intracrine functions of VEGF-A. Biomolecules 11:128. 10.3390/biom11010128 33478167 10.3390/biom11010128 PMC7835749

[CR182] Wong TY, Cheung CMG, Larsen M, Sharma S, Simó R (2016) Diabetic retinopathy. Nat Rev Dis Primers 2:16012 10.1038/nrdp.2016.12 27159554

[CR183] Wu Y, Li X, Fu X, Huang X, Zhang S, Zhao N, Ma X, Saiding Q, Yang M, Tao W (2024) Innovative nanotechnology in drug delivery systems for advanced treatment of posterior segment ocular diseases. Adv Sci 11:2403399. 10.1002/advs.202403399 10.1002/advs.202403399 PMC11348104 39031809

[CR184] Wykoff CC (2017) Impact of intravitreal pharmacotherapies including antivascular endothelial growth factor and corticosteroid agents on diabetic retinopathy. Curr Opin Ophthalmol 28:213–218 28376510 10.1097/ICU.0000000000000364

[CR185] Wykoff CC, Khurana RN, Nguyen QD, Kelly SP, Lum F, Hall R, Abbass IM, Abolian AM, Stoilov I, To TM (2021) Risk of blindness among patients with diabetes and newly diagnosed diabetic retinopathy. Diabetes Care 44:748–756 33472864 10.2337/dc20-0413 PMC7896265

[CR186] Xie T, Chen X, Chen W, Huang S, Peng X, Tian L, Wu X, Huang Y (2021) Curcumin is a potential adjuvant to alleviates diabetic retinal injury via reducing oxidative stress and maintaining Nrf2 pathway homeostasis. Front Pharmacol 12:796565 34955862 10.3389/fphar.2021.796565 PMC8702852

[CR187] Xie Y, Wang C (2023) Herb–drug interactions between Panax notoginseng or its biologically active compounds and therapeutic drugs: a comprehensive pharmacodynamic and pharmacokinetic review. J Ethnopharmacol 307:116156 36754189 10.1016/j.jep.2023.116156

[CR188] Xiong X-f, Wei L, Xiao Y, Han Y-C, Yang J, Zhao H, Yang M, Sun L (2020) Family history of diabetes is associated with diabetic foot complications in type 2 diabetes. Sci Rep 10:17056 33051498 10.1038/s41598-020-74071-3 PMC7555504

[CR189] Xu K, Qian H, Zou M (2020) Triamcinolone acetonide combined with aminoguanidine inhibits inflammation and oxidative stress, improves vascular endothelial and retinal function and reduces VEGF expression in diabetic retinopathy patients. Exp Ther Med 19:2519–2526 32256730 10.3892/etm.2020.8478 PMC7086164

[CR190] Xu M, Fan R, Fan X, Shao Y, Li X (2023) Progress and challenges of anti-VEGF agents and their sustained-release strategies for retinal angiogenesis. Drug Des Dev Ther 3241–3262. 10.2147/DDDT.S383101 10.2147/DDDT.S383101 PMC9512290 36172053

[CR191] Xu X, He M, Zhao G, Liu X, Liu X, Xu H, Cheng Y, Jiang Y, Peng Q, Shi J (2024) The association of dietary diversity with hyperuricemia among community inhabitants in Shanghai, China: a prospective research. Nutrients 16:2968 39275283 10.3390/nu16172968 PMC11397405

[CR192] Xu Y, Yu J (2024) Allicin mitigates diabetic retinopathy in rats by activating phosphatase and tensin homolog-induced kinase 1/parkin-mitophagy and inhibiting oxidative stress-mediated NOD-like receptor family pyrin domain containing 3 inflammasome. J Physiol Investig 67:215–224 39206781 10.4103/ejpi.EJPI-D-24-00039

[CR193] Yang F (2017) Clinical effect of pills of six ingredients with Rehmannia combined with Ginkgo biloba on prevention and treatment of early retinopathy in type 2 diabetes mellitus patients. Int Eye Sci 12:1127–1129

[CR194] Yang F, Yu J, Ke F, Lan M, Li D, Tan K, Ling J, Wang Y, Wu K, Li D (2018) Curcumin alleviates diabetic retinopathy in experimental diabetic rats. Ophthalmic Res 60:43–54 29597206 10.1159/000486574

[CR195] Yang J, Liu Z (2022) Mechanistic pathogenesis of endothelial dysfunction in diabetic nephropathy and retinopathy. Front Endocrinol 13:816400 10.3389/fendo.2022.816400 PMC9174994 35692405

[CR196] Yang J, Miao X, Yang F-J, Cao J-F, Liu X, Fu J-L, Su G-F (2021) Therapeutic potential of curcumin in diabetic retinopathy. Int J Mol Med 47:1–12 10.3892/ijmm.2021.4908 PMC7949626 33693955

[CR197] Yang X, Huo F, Liu B, Liu J, Chen T, Li J, Zhu Z, Lv B (2017) Crocin inhibits oxidative stress and pro-inflammatory response of microglial cells associated with diabetic retinopathy through the activation of PI3K/Akt signaling pathway. J Mol Neurosci 61:581–589 28238066 10.1007/s12031-017-0899-8

[CR198] Yang X, Yu X-W, Zhang D-D, Fan Z-G (2020) Blood-retinal barrier as a converging pivot in understanding the initiation and development of retinal diseases. Chin Med J 133(21):2586–2594. 10.1097/CM9.0000000000001015 10.1097/CM9.0000000000001015 PMC7722606 32852382

[CR199] Yao Q, Yang Y, Lu X, Zhang Q, Luo M, Li PA, Pan Y (2018) Lycium barbarum polysaccharides improve retinopathy in diabetic Sprague-Dawley rats. Evid-Based Complement Alternat Med 2018:7943212 30581486 10.1155/2018/7943212 PMC6276478

[CR200] Yau JW, Rogers SL, Kawasaki R, Lamoureux EL, Kowalski JW, Bek T, Chen S-J, Dekker JM, Fletcher A, Grauslund J (2012) Global prevalence and major risk factors of diabetic retinopathy. Diabetes Care 35:556–564 22301125 10.2337/dc11-1909 PMC3322721

[CR201] Ye X, Fung NSK, Lam WC, Lo ACY (2024) Nutraceuticals for diabetic retinopathy: recent advances and novel delivery systems. Nutrients 16:1715 38892648 10.3390/nu16111715 PMC11174689

[CR202] Yu H, Wark L, Ji H, Willard L, Jaing Y, Han J, He H, Ortiz E, Zhang Y, Medeiros DM (2013) Dietary wolfberry upregulates carotenoid metabolic genes and enhances mitochondrial biogenesis in the retina of db/db diabetic mice. Mol Nutr Food Res 57:1158–1169 23505020 10.1002/mnfr.201200642 PMC3838638

[CR203] Yumnamcha T, Guerra M, Singh LP, Ibrahim AS (2020) Metabolic dysregulation and neurovascular dysfunction in diabetic retinopathy. Antioxidants 9:1244 33302369 10.3390/antiox9121244 PMC7762582

[CR204] Yurievna IN, Victorovich KD (2022) Decision-making support system for the personalization of retinal laser treatment in diabetic retinopathy. Кoмпьютepнaя Oптикa 46:774–782

[CR205] Zhang C-J, Jin Z-B, Zhang C-J, Jin Z-B (2023) Homeostasis and dyshomeostasis of the retina. Curr Med 2. 10.1007/s44194-023-00021-6

[CR206] Zhang J, Zhang J, Zhang C, Zhang J, Gu L, Luo D, Qiu Q (2022a) Diabetic macular edema: current understanding, molecular mechanisms and therapeutic implications. Cells 11:3362 36359761 10.3390/cells11213362 PMC9655436

[CR207] Zhang L, Chu W, Zheng L, Li J, Ren Y, Xue L, Duan W, Wang Q, Li H (2020) Zinc oxide nanoparticles from Cyperus rotundus attenuates diabetic retinopathy by inhibiting NLRP3 inflammasome activation in STZ-induced diabetic rats. J Biochem Mol Toxicol 34:e22583 32692483 10.1002/jbt.22583

[CR208] Zhang W, Geng J, Sang A (2022b) Effectiveness of panretinal photocoagulation plus intravitreal anti-VEGF treatment against PRP alone for diabetic retinopathy: a systematic review with meta-analysis. Front Endocrinol 13:807687 10.3389/fendo.2022.807687 PMC9004461 35422768

[CR209] Zhang W, Liu Y, Sang A (2022c) Efficacy and effectiveness of anti-VEGF or steroids monotherapy versus combination treatment for macular edema secondary to retinal vein occlusion: a systematic review and meta-analysis. BMC Ophthalmol 22:472 36474156 10.1186/s12886-022-02682-7 PMC9727869

[CR210] Zhang Z, Deng C, Paulus YM (2024) Advances in structural and functional retinal imaging and biomarkers for early detection of diabetic retinopathy. Biomedicines 12:1405 39061979 10.3390/biomedicines12071405 PMC11274328

[CR211] Zhao M, Chandra A, Xu J, Li J (2023) Factors related to postoperative vitreous hemorrhage after small-gauge vitrectomy in proliferative diabetic retinopathy patients. BMC Ophthalmol 23:215 37189104 10.1186/s12886-023-02940-2 PMC10183694

[CR212] Zhao Y, Chen Y, Yan N (2024) The role of natural products in diabetic retinopathy. Biomedicines 12:1138 38927345 10.3390/biomedicines12061138 PMC11200400

[CR213] Zheng H-S, Lan Y-Q, Zhong X-W, Zhou H-S, Xu J-Y (2022) Effects of curcumin nanoparticles on proliferation and VEGF expression of human retinal pigment epithelial cells. Int J Ophthalmol 15:905 35814903 10.18240/ijo.2022.06.07 PMC9203465

[CR214] Ziamajidi N, Abbasalipourkabir R, Lotfi F, Goodarzi MT (2022) Aqueous garlic extract alleviates oxidative stress and inflammation in retinal tissue of rats with diabetes type 2. J Adv Med Biomed Res 30:138–145

